# A new strain of *Rhodococcus indonesiensis* T22.7.1^T^ and its functional potential for deacetylation of chitin and chitooligsaccharides

**DOI:** 10.3389/fmicb.2024.1427143

**Published:** 2024-07-24

**Authors:** Junjie Xie, Doudou Yin, Junchao Ou, Bo Lu, Siming Liao, Dengfeng Yang, Hongyan Zhang, Naikun Shen

**Affiliations:** ^1^Guangxi Key Laboratory for Polysaccharide Materials and Modifications, School of Marine Sciences and Biotechnology, Guangxi Minzu University, Nanning, China; ^2^Guangxi Key Laboratory of Marine Natural Products and Combinatorial Biosynthesis Chemistry, Guangxi Beibu Gulf Marine Research Center, Guangxi Academy of Sciences, Nanning, China

**Keywords:** *Rhodococcus indonesiensis*, chitin deacetylase, fermentation optimization, enzyme activity, chitin oligosaccharides

## Abstract

**Introduction:**

Chitin, abundant in marine environments, presents significant challenges in terms of transformation and utilization. A strain, T22.7.1^T^, with notable chitin deacetylation capabilities, was isolated from the rhizosphere of *Acanthus ebracteatus* in the North Sea of China. Comparative 16S rDNA sequence analysis showed that the new isolate had the highest sequence similarity (99.79%) with *Rhodococcus indonesiensis* CSLK01-03^T^, followed by *R. ruber* DSM 43338^T^, *R. electrodiphilus* JC435^T^, and *R. aetherivorans* 10bc312^T^ (98.97%, 98.81%, and 98.83%, respectively). Subsequent genome sequencing and phylogenetic analysis confirmed that strain T22.7.1^T^ belongs to the *R. indonesiensis* species. However, additional taxonomic characterization identified strain T22.7.1^T^ as a novel type strain of *R. indonesiensis* distinct from CSLK01-03^T^.

**Methods:**

This study refines the taxonomic description of *R. indonesiensis* and investigates its application in converting chitin into chitosan. The chitin deacetylase (*Ri*CDA) activity of strain T22.7.1^T^ was optimized, and the enzyme was isolated and purified from the fermentation products.

**Results:**

Through optimization, the *Ri*CDA activity of strain T22.7.1^T^ reached 287.02 U/mL, which is 34.88 times greater than the original enzyme’s activity (8.0 U/mL). The natural CDA enzyme was purified with a purification factor of 31.83, and the specific activity of the enzyme solution reached 1200.33 U/mg. *Ri*CDA exhibited good pH and temperature adaptability and stability, along with a wide range of substrate adaptabilities, effectively deacetylating chitin, chitooligosaccharides, N-acetylglucosamine, and other substrates.

**Discussion:**

Product analysis revealed that *Ri*CDA treatment increased the deacetylation degree (DD) of natural chitin to 83%, surpassing that of commercial chitosan. Therefore, *Ri*CDA demonstrates significant potential as an efficient deacetylation tool for natural chitin and chitooligosaccharides, highlighting its applicability in the biorefining of natural polysaccharides.

## Graphical abstract

This paper identified a new strain of marine actinomycete that produces chitin deacetylase, which offers new insight into the biotransformation of chitin and chitooligosaccharides.

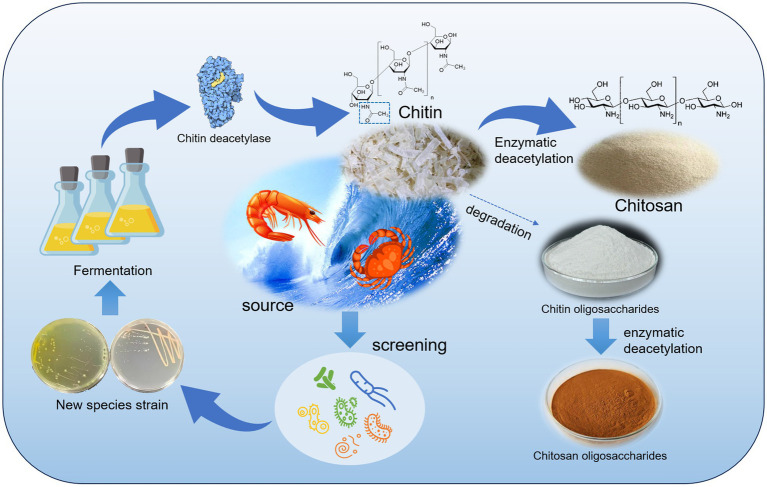

## Introduction

Chitin is a natural polysaccharide composed of N-acetyl-D-glucosamine (NAG) units associated with β-1,4 glycosidic bonds. It is the second largest natural polysaccharide in existence and is commonly found in the cell walls of fungi and algae, as well as in the exoskeletons of insects and shells of crustaceans (Hahn et al., 2020; Fernando et al., 2021; Pakizeh et al., 2021; Shao et al., 2023). Natural chitin is insoluble in water and common reagents due to the N-acetyl groups present in its linear macromolecules, which create intermolecular hydrogen bonds that enhance its structural stability and hydrophobicity (Jang et al., 2004; Rinaudo, 2006). This characteristic limit the potential uses of chitin. However, when chitin is fully or partially deacetylated, it can be converted into chitosan. Limits the exploitation of chitin. The solubility of chitosan is enhanced, and the deacetylated -NH_2_ ions in solution to become alkaline (Kumar et al., 2004; Ogawa et al., 2004). Chitosan has a wide range of applications in the medical (Baharlouei and Rahman, 2022; Saeedi et al., 2022), food (Mujtaba et al., 2019), cosmetic (Triunfo et al., 2021), flocculant (Lichtfouse et al., 2019), and plant promotion (Shahrajabian et al., 2021) industries due to its unique structural properties and biological activities. Deacetylation of chitin not only improves its solubility but also results in deacetylated products with diverse biological activities and application potential.

Chitosan oligosaccharides (COSs), which are derivatives of chitin and chitosan, have attracted significant interest due to their low molecular weight and high solubility (Lodhi et al., 2014). COSs are characterized by an average molecular weight (Mw) of less than 3.9 kDa and typically contain fewer than 20 monomeric units per polymer chain. These compounds include chitosan oligosaccharides (CSOs) and chitin oligosaccharides (CTOs). CSO can be obtained through acid hydrolysis, physical degradation, or enzymatic hydrolysis of chitosan to break the glycosidic bonds of the polymers and remove the N-acetyl group (Hamer et al., 2015; Benchamas et al., 2021). Enzymatic deacetylation of CTO can also produce CSO. In theory, CTO should have 100% DD, whereas CSO should have no acetylation (Yang et al., 2022a). However, due to variations in the preparation methods, many COSs contain both N-acetylglucosamine and glucosamine, resulting in partially acetylated chitooligosaccharides (paCOSs) (Yin et al., 2016). paCOS has potent biological properties and numerous potential applications. Its biological activity is believed to depend on its structure, including its degree of polymerization (DP) and degree of acetylation (DA), as well as its pattern of acetylation (PA) (Naqvi et al., 2016; Naveed et al., 2019; Bonin et al., 2020; Miao et al., 2021). Previous studies have demonstrated the antimicrobial, anti-inflammatory, antioxidant, and immunomodulatory activities of chitosan and its derivatives, highlighting their potential applications in the fields of food, medicine, and cosmetics (Ouyang et al., 2017; Lin et al., 2022; Sun et al., 2022; Chen et al., 2023).

Despite the considerable potential of chitin, its deacetylation process presents several challenges. Chitin is known for its insolubility in water and most organic solvents, which makes achieving uniform deacetylation difficult. Traditionally, chemical hydrolysis has been utilized for preparing chitosan and COS, but this method lacks control over the structural properties of the products, involves the use of large quantities of strong acids and bases and is not environmentally friendly (Lodhi et al., 2014; Naveed et al., 2019; Arnold et al., 2020). In recent years, there has been an increased focus on the enzymatic deacetylation of chitin (CDA), which offers precise control over the DD in chitosan and the production of specific PA (paCOS) products. Additionally, enzymatic deacetylation is considered gentler, greener, more efficient, and more sustainable (Schmitz et al., 2019; Charoenpol et al., 2023; Cheng et al., 2023; Zhang et al., 2023).

The currently studied CDA enzymes are increasingly recognized as valuable tools for the production of fully defined paCOSs due to their selectivity in targeting specific regions. However, there is a lack of research on CDA production by strains of the genus *Rhodococcus* (Aragunde et al., 2018; Grifoll-Romero et al., 2018; Bai et al., 2020; Chai et al., 2020; Cheng et al., 2023). Previous studies have suggested that *Rhodococcus* spp. have the potential to produce highly active CDA. However, there are a limited number of isolated and characterized model members of the genus *Rhodococcus* from the environment, with only 97 known species. Furthermore, there are few reports on chitin deacetylation activity among these model species.

Currently, chitinase and chitosanase are commercially available, but chitin deacetylase remains unavailable. Numerous chitin deacetylases from various sources, including bacteria, fungi, and insects, have been identified and characterized, with particular attention given to their structure, mechanism of action, and substrate specificity. Studies have demonstrated that CDAs from different species exhibit diverse biological activities, with variations in their substrates and reaction conditions (Aragunde et al., 2018). Although recent research has reported on CDA enzyme activity in chitin deacetylation by strains from several genera, such as *Acinetobacter* (Yang et al., 2022a, 2023), *Bacillus* (Liang et al., 2022; Zhang et al., 2023), *Arthrobacter* (Ding et al., 2021), *Microbacterium* (Yang et al., 2022a) and *Streptomyce* (Yin et al., 2022), these CDA activities are either low or confined to narrow substrate specificities against chitin or CTO. Consequently, numerous studies have been conducted to optimize the reaction conditions, aiming to enhance the efficiency and yield of enzymatic deacetylation (Sun et al., 2014; Yang et al., 2022a).

The currently studied CDA enzymes are increasingly recognized as valuable tools for the production of fully defined paCOSs due to their selectivity in targeting specific regions (Aragunde et al., 2018; Bonin et al., 2020; Biniek-Antosiak et al., 2022; Cheng et al., 2023). However, there is a lack of research on CDA production by strains of the genus *Rhodococcus*. Previous studies have suggested that *Rhodococcus* spp. have the potential to produce highly active CDA (Sun et al., 2014; Ma et al., 2020). However, there are a limited number of isolated and characterized model members of the genus Rhodococcus from the environment, with only 97 known species. Furthermore, there are few reports on chitin deacetylation activity among these model species.^1^

In contrast, the currently available *Rhodococcus erythropolis* strain HG05, which has undergone complex fermentation optimization, only exhibited an enzyme activity of 238.89 U/mL (Sun et al., 2014). In this study, we identified a CDA-producing *Rhodococcus* strain, T22.7.1, which is suspected to be a new species. We investigated its taxonomic status using various methods and optimized the conditions for the production of its enzymes. Through simple process optimization, we achieved a much higher enzyme activity than what has been reported for existing *R. erythropolis* strains. We further isolated and obtained the purified CDA natural enzyme and investigated its enzymatic properties. This allowed us to determine the broad range of substrates it acts on and the characteristics of its catalytic products. Our findings demonstrate that strain T22.7.1 is a more efficient and highly active CDA-producing strain for the deacetylation of chitin and CTO. Additionally, we used genome mining and product characterization to clarify the functional potential of the Rhodococcus strain T22.7.1 in chitin biotransformation. Our study provides a valuable enzymatic tool for the environmentally friendly and efficient production of biologically active paCOS.

## Materials and methods

### Chemicals and reagents

Chitin was purchased from Aladdin Biochemical Technology Co. Ltd. (Shanghai, China); chitosan and chitosan oligosaccharides were purchased from Yuanye Biotechnology (Shanghai, China); N-acetyl-D-glucosamine was purchased from Solebo (Beijing, China). The colloidal chitin was prepared as the previously reported method (Pareek et al., 2013). p-Nitroacetanilide was purchased from Bailingwei Technology Co., Ltd. (Beijing, China). Bacterial genomic DNA extraction kit and PCR product/gel extraction kit were purchased from Tigen Biochemicals (Beijing, China); acetic acid assay kit was purchased from Solepol (Beijing, China); Millex^®^-GP 0.22um disposable injectable membranes and Ultra-15 centrifugal filters were purchased from Merck-Millipore (Beijing, China). Superdex 75 Increase 10/300 GL and Q Sepharose High Performance were both purchased from GE Healthcare (Sweden). Molecular weight markers (14.4 to 116 kDa) were purchased from Sangon Biotech Co. N-acetyl chito-oligomers ((GlcNAc)_1–6_) were purchased from Qingdao BZ Oligo Biotech Co. The chito-oligomers mixture was purchased from TCI (Shanghai, China). All other chemicals and reagents were analytically pure.

### Strain isolation and preservation

The strain T22.7.1^T^ with obvious deacetylation ability was isolated from the rhizosphere of *Acanthus ebracteatus* of Guangxi Beihai beach (N21.624, E108.909), China. The strain was purified by dilution plating method and streak inoculated on chitin deacetylation functional screening medium (g/L: p-nitroacetanilide 2; colloidal chitin 2; K_2_HPO_4_ 0.7; KH_2_PO_4_ 0.3; MgSO_4_ 0.5; NaCl 0.1; agar 20; pH 7.0) (Zhang et al., 2023). Strain T22.7.1^T^ was inoculated by streaking on ISP2 medium and collected after 72 h of purification incubation at 28°C, and stored in 30% (v/v) glycerol at −80°C (Xie et al., 2023a). The strain has been deposited in the Marine Culture Collection of China (MCCC) and Japan Microbe Division (JCM) under the following numbers: MCCC 1 K08698 and JCM 36625, respectively.

### Extraction, sequencing and phylogenetic analysis of 16S rDNA gene and genome

The genome of strain T22.7.1^T^ was extracted using the Genomic DNA Extraction Kit (TIANGEN) and the 16S rRNA gene was amplified as previously described by Li *et al* (Li et al., 2007). 16S rRNA gene sequencing was performed by Wuhan AOKE DINGSHENG BIOTECHNOLOGY CO., LTD.^2^ using the Sanger method. The 16S rDNA sequence of strain T22.7.1^T^ was identified and analyzed using BLAST^3^ and EzBioCloud^4^ server databases, and online comparison analysis was performed to obtain the 16S rDNA sequences with the highest degree of similarity to the strain. The 16S rDNA sequences of strain T22.7.1^T^ and its close relatives were analyzed for multiple comparisons and sequence similarity levels were calculated using the Clustal W program of MEGA 11 (Tamura et al., 2021); neighbor-joining (Saitou and Nei, 1987), maximum likelihood (Felsenstein, 1981), and maximum parsimony (Fitch, 1971) were used to construct a phylogenetic tree using a self-expansion value of 1,000 resampling replicates, respectively (Felsenstein, 1985). The whole genome draft of strain T22.7.1^T^ was sequenced by Shanghai Majorbio Bio-Pharm Technology Co., Ltd.^5^ using Illumina HiSeq X10 platform. The complete genome sequence was assembled *de novo* by SPAdes 3.13.0 using the online patric server (Bankevich et al., 2012; Wattam et al., 2017). The whole genome sequence of strain T22.7.1^T^ was phylogenetically analyzed using the Type Strain Genome Server (https://tygs.dsmz.de) (Meier-Kolthoff and Göker, 2019), and by the genome blast distance phylogeny (GBDP) based FastME 2.1.6.1. program to construct a phylogenetic tree (Lefort et al., 2015).

The complete 16S rDNA sequence (1,473 bp) and the whole genome sequence (WGS) of strain T22.7.1^T^ were submitted to the GenBank database with the DDBJ/EMBL/GenBank accession numbers OQ976993 and JASKMB000000000, respectively.

### Culture and phenotypic characterization

Cell morphology was visualized by field emission scanning electron microscopy (Carl Zeiss Supra 55 Sapphire) after 2 weeks of incubation at 28°C on ISP 2 agar. Culture characteristics were examined after 2 weeks of incubation at 28°C on ISP 1–7 agar (Shirling and Gottlieb, 1966), Czapek agar (CA) (Waksman, 1967), tryptic soy agar (TSA; Difco), starch ammonia agar (SAA) (Barreiro et al., 2021), and lysogeny broth agar (LB, Difco). Colony color was determined according to the ISCC-NBS color scale (No. 2106) (Kelly, 1964). Cell motility was determined according to the previous method (Leifson, 1960). Temperature (10–60°C at 5°C intervals), pH [4–11 at 1 interval, buffer systems used refer to previous descriptions (Xie et al., 2023a)] and NaCl (0–14% w/v at 2% intervals) tolerance assays were tested on ISP 2 medium, incubated at 28°C for 2 weeks each and continuously monitored (Ramaprasad et al., 2015; Maiti and Mandal, 2019; Maiti and Mandal, 2020).

### Chemical taxonomic analysis

Biomass for chemical composition analysis was obtained and lyophilized as the methods described by us previously (Xie et al., 2023a,b)Whole cell hydrolysate sugars and characteristic amino acids of strain T22.7.1^T^ were analyzed by thin layer chromatography (TLC) (Lechevalier et al., 1971; Staneck and Roberts, 1974). Extraction of polar lipids and methylnaphthoquinone was carried out following the method described by Minnikin et al. (1984), and the polar lipids were analyzed by spreading according to the two-dimensional TLC method described by Goodfellow et al. (1980). The main respiratory quinone types of strain T22.7.1^T^ were analyzed by HPLC method (Kroppenstedt, 1982). The cellular fatty acid composition was tested by the Marine Conservation Center of China (Xiamen, PR China) using the Sherlock Microbial Identification System, and the fatty acid methyl esters were quantified using the TSBA 6.0 database (Sasser, 1990).

### Comparative genomic analysis

Comparative analysis of the genomic data of strain T22.7.1^T^ and the species most relevant to its development was performed based on phylogenetic relationships of whole genome sequence reconstruction. Genomic coding sequences (CDSs) of the strain genome were annotated using the Rapid Annotation Tool of the Subsystems Technology server (RAST) (Aziz et al., 2008). Comparative analysis of homology clusters of strain T22.7.1^T^ and its close relatives was performed using OrthoVenn3 (Sun et al., 2023). The average nucleotide identity (ANI) value between strain T22.7.1^T^ and its closer relatives was calculated using JSpecies Web Server based on the Blast+ method (Richter et al., 2016). Digital DNA–DNA hybridization (dDDH) values based on the strain’s whole genome sequence, Genome-to-Genome Distance and Difference %G + C were calculated using the Genome-to-Genome Distance Calculator (GGDC 3.0; http://ggdc.dsmz.de) and the recommended dDDH results of Equation 2 were adopted (Meier-Kolthoff et al., 2022).

### Genome mining and metabolic system analysis

Coding sequences (CDS) in the genome were predicted using Prodigal, which was used to predict chromosomal genes, and GeneMarkS, which was used to predict plasmid genes. The tRNAs and rRNAs contained in the genome were predicted using tRNAscan-SE^6^ and Barrnap,^7^ respectively. The predicted CDSs were annotated using Non-Redundant Protein Database (NR), Swiss-Prot (Bairoch and Apweiler, 2000), Pfam (Mistry et al., 2021), evolutionary genealogy of genes: Non-supervised Orthologous Groups (eggNOG) (Jensen et al., 2008), Gene Ontology (GO), and Kyoto Encyclopedia of Genes and Genomes (KEGG) (Kanehisa and Goto, 2000) databases using Blast2Go, Diamond and HMMER3 as sequence alignment tools (Sun et al., 2016; Xiao et al., 2022). Briefly, the query proteins were aligned with the databases to obtain the gene annotations corresponding to the most optimal matches (E-value <10^−5^).

The metabolic system analysis of the strains included annotation of carbohydrate-activating enzymes and secondary metabolite synthesis gene cluster analysis. HMMER3 was used to derive annotation information corresponding to carbohydrate-active enzymes (CAZymes) based on whole-genome sequence deduced to protein sequence comparison to the CAZymes database (CAZy, http://www.cazy.org/) (Drula et al., 2022). The screening condition was E-value <1e^−5^. On the other hand, the strain genomes were rapidly identified, annotated and analyzed using antiSMASH 7.0 to know the functional potential of microorganisms to synthesize secondary metabolites (Blin et al., 2023).

### Optimization of fermentation conditions

Strain T22.7.1 was aseptically inoculated into ISP2 liquid medium fermented at 28°C, 180 rpm, and shaking flask for 48 h as seed solution. The fermented seed liquid was inoculated into a sterilized 30 mL basal fermentation medium at a 1% (v/v) inoculum volume. It was then placed in a shaker at 28°C and 200 rpm for 72 h to cultivate the CDA enzyme through liquid fermentation under basal conditions.

The fermented seed liquid was inoculated into the sterilized 30 mL (20% loading, v/v) basal fermentation medium (g/L, KH_2_PO_4_ 0.3, K_2_HPO_4_ 0.7, (NH_4_)_2_SO_4_ 2, glucose 2.5, yeast extract 5) at 1% (v/v) inoculum volume. It was then placed in a shaker at 28°C and 200 rpm for 72 h to cultivate the CDA enzyme through liquid fermentation under basal conditions. To optimize the medium composition and culture conditions, both a one-way experiment and an orthogonal design were employed. The one-way experiments focused on optimizing one ingredient at a time in the basic fermentation medium. Each condition was optimized based on the previous one. Several carbon sources (chitin, glucose, maltose, fructose, sucrose, and soluble starch), nitrogen sources (yeast extract, beef paste, peptone, soybean extract, ammonium chloride, and urea), and inorganic salts (CaCl_2_, MnSO_4_, FeSO_4_, KCl, NaCl, and MgSO_4_) were selected as candidate components for the medium composition. The basal medium was used as a control for optimizing the medium composition. Furthermore, the initial pH of the medium and fermentation conditions (temperature, fermentation time, loading volume, and inoculum volume) were optimized in a similar manner.

### CDA enzyme activity assay

The colorimetric method (p-nitroacetanilide as substrate) used to assay CDA enzyme activity was slightly modified from a previous description (Liping, 2008). The standard curve was obtained by determining the correspondence between the absorbance value of the reaction substrate p-nitroacetanilide deacetylation product p-nitroaniline at 400 nm, OD_400_, and the concentration of the product. In each numbered 1 mL capacity eppendorf centrifuge tube (EP tube), 0.3 mL of phosphate buffer solution at pH 7 concentration of 0.05 mol/L was added, and after a water bath at 50°C for 5 min, 0.1 mL of p-nitroacetanilide solution at concentration of 200 mg/L and 0.1 mL of crude enzyme solution were added respectively, and 0.1 mL of the control colorimetric tube was added. Add 0.1 mL of p-nitroacetanilide solution, 0.1 mL of crude enzyme solution after inactivation in boiling water bath, add the crude enzyme solution after inactivation in boiling water bath; keep it at 50°C for 15 min, then put it into boiling water bath for 5 min, cool it down, and then make it into 1 mL with distilled water, shake it well and centrifugate at 10000 rpm for 10 min, and then take supernatant to determine the absorbance value at 400 nm wavelength. The enzyme activity was calculated against the standard curve of p-nitroaniline.

The enzyme activity unit (U) of CDA was defined as the amount of enzyme required to catalyse the production of 1 μg of p-nitroaniline from the substrate by chitin deacetylase per hour under the above conditions (Sun et al., 2014). The formula for the standard curve used to calculate the p-nitroaniline content of the product, constructed according to the above method, is given below:


$$y=0.1047x+0.0457\;\left(R^{2}=0.9999\right)$$

HPLC method used to the different deacetylation activities of strain CDA on chitin, chitosan, CTO, CSO, and NAG were tested by using an acetic acid content assay kit and by high-performance liquid chromatography (HPLC) detection technique (Bai et al., 2020; Cheng et al., 2023). The monosaccharide and oligosaccharide substrates were dissolved in phosphate buffer (50 mM, pH 7.0) and 500 μL of substrate solution was taken and 500 μL of enzyme solution was configured to form a 1 mL reaction system, which was placed in a 50°C water bath for 1 h and then boiled for 10 min to terminate the reaction. Insoluble polysaccharide substrate with enzyme solution and phosphate buffer solution to form a 1 mL reaction system was placed in 37°C shaker 180 rpm shaking 3 h after boiling for 10 min to terminate the reaction. The reaction mixture was centrifuged at 10000 rpm for 20 min, and the supernatant was filtered through a 0.22 μm membrane and used as the sample for HPLC analysis under the following conditions: detection on an Ultimate AQ-C18 column (5 μm, 250 × 4.6 mm) at a column temperature of 30°C and a UV filter at a wavelength of 210 nm, and the flow rate was 0.4 mL/min. Each acetic acid detection was repeated three times. The concentration of acetic acid in the samples was calculated using standard curves generated from 175, 87.5, 17.5, 8.75, 1.75 and 0.875 μmol/mL of acetic acid with the following equation:


$$y=40.557x-31.508\;\left(R^{2}=0.9998\right)$$

The amount of enzyme required to release 1 μmol of acetic acid per minute under the above reaction conditions was defined as one unit of enzyme activity (U) (Bai et al., 2020).

### Enzyme purification, enzymatic properties and product characterization

The purification of CDA was carried out with slight modification of the method described by a previous author (Chai et al., 2020). The strain was cultured in the optimized fermentation medium and conditions to produce the CDA enzyme. The fermentation product was subjected to ultrasonic cell disruption in an ice bath, followed by centrifugation at 12000 rpm for 20 min. The resulting supernatant was considered the crude enzyme broth. Ammonium sulfate powder was added to the crude enzyme broth in an ice bath until a saturation level of 75% was reached. The precipitate was collected by centrifugation at 12000 rpm for 20 min and reconstituted with ultrapure water; residual ammonium sulphate was removed and replaced with 10 mM phosphate buffer (pH 7.0) using a 30 kDa ultrafiltration tube. The crude enzyme solution was loaded onto a Q Sepharose High Performance gel chromatography column (16 mm × 40 cm), pre-equilibrated with 10 mM sodium phosphate buffer pH 7.0, and then eluted with a linear gradient of 0–1.0 M NaCl. The eluted components were UV-detected at 280 nm as described above, and the eluted peak components were collected into different EP tubes in 1 mL volumes. The enzyme activity in each collection tube was tested using a colorimetric method, and based on the test results, the eluent with enzyme activity was mixed and introduced onto a Superdex 75 Increase 10/300 GL column (10 mm × 30 cm). The column was eluted with 50 mM Tris–HCl (pH 7.5) containing 0.15 M NaCl, and the elution components were monitored at 280 nm wavelength, and the elution peak components were collected into different EP tubes in 0.5 mL volumes. After CDA enzyme activity was tested by colorimetric method, the collected solution with CDA activity was mixed and replaced with a solution of 10 mM sodium phosphate buffer through a 30 kDa ultrafiltration tube. Sodium dodecyl sulphate polyacrylamide gel electrophoresis (SDS-PAGE) was performed using 10% w/v polyacrylamide gel (Pareek et al., 2013). Proteins in the polyacrylamide gel were color developed with Coomassie Brilliant Blue G250.

#### Enzymatic property test

Enzyme activity was determined at temperatures ranging from 30 to 70°C (at 10°C intervals) using a phosphate buffer solution at pH 7.0. The reaction solution was maintained at the specified temperatures for 0–10 h, and enzyme activity was measured every 2 h. Enzyme activity at 0 h at each temperature was taken as 100% to study temperature stability. Different pH values were tested at 50°C in a water bath (pH 5–9 at intervals of 1, 50 mM citrate buffer 5–6, 50 mM phosphate buffer 6–8 and Tris- HCl buffer 8–9.) to assess CDA enzyme activity and stability. Metal salts (Ca^2+^, Mg^2+^, Mn^2+^, Zn^2+^, Cu^2+^, Fe^3+^, Fe^2+^, Ni^2+^, Na^+^, K^+^) and chemical reagents (EDTA, SDS, PSMF, Tween 20, Tween 80) with the same ionic concentration were added to the reaction system to reach the final concentration of 1 mM for each metal ion and 5 mM for each chemical reagent, respectively and enzyme activity assay was carried out without adding any other substance to the reaction system as a control, and three replicates were set up for each set of experiments.

#### Characterization of the deacetylation products of different substrates

The deacetylation products of *Ri*CDA-treated CTO were analysed using the TLC method (Kang et al., 2014; Yang et al., 2022a). The reaction products were analysed with a TLC silica gel plate (Merck KGaA, Darmstadt, Germany), and the solvent for chromatography development was n-butanol/anhydrous ethanol/water (5,3,2, v/v). The spots of sugar on the plate were observed by spraying the color developer (1.0 g of diphenylamine dissolved in 50.0 mL of acetone, followed by the gradual addition of 1.0 mL of aniline and 5.0 mL of phosphoric acid) and baking the plate for 5 min at 85°C. On the other hand, chitin oligosaccharides (DP 1–5, TCI) were separated by TLC method, the color-developed spots were used as templates to scrape the silica gel at the position of specific shift values, and the scraped silica gel was dissolved in 1 mL of ultrapure water, mixed and filtered through a 0.22 μm membrane, and then the aqueous phase after extraction was repeated twice using methylene chloride to collect the aqueous phase after extraction as a single polymerization degree of the chitin oligosaccharides samples were used as substrates for subsequent *Ri*CDA treatment. SEM and FTIR of chitin deacetylation products: 0.2 g of chitin powder and 1 mL of *Ri*CDA enzyme solution were added to a 2 mL EP tube, mixed, and then placed at 37°C, 180 rpm shaker reaction for 12 h, boiled for 10 min to terminate the reaction, and centrifuged at 10000 rpm for 10 min, and then the precipitate was washed with deionized water for two times, and then dried at 50°C for 24 h as the samples for SEM and FTIR tests. SEM and FT-IR tests were performed using water-treated chitin powder as a control. Photographs of the surface microforms of the samples were taken using a Nippon Electron JSM-IT100 scanning electron microscope with a magnification of 2000, 5,000, and 10,000, respectively, following the previous methods (Li et al., 2019; Yang et al., 2022a). Dried potassium bromide and the samples were weighed according to the ratio of 120:1, and after grinding and mixing, 0.2 g of mixture was weighed and made into thin slices by using a hand-operated tableting press (769YP -15A) to make thin slices and FT-IR spectroscopy (Nicolet-iS10) was used to scan the region with a wave number of 400–4,000 cm^−1^. The intensity ratio of the characteristic absorption peaks with wave numbers of 1,655 cm^−1^ (amide I) and 3,450 cm^−1^ (OH group) correlated linearly with the degree of deacetylation of the chitin samples, which was calculated using the following equation (Peh et al., 2000).


$$D.D=\left(1-\frac{A_{1655}/A_{3450}}{1.33}\right)\times 100\%$$

### Data analysis and graphing software

All trials were conducted in triplicate and data are expressed as mean ± standard deviation. One-way ANOVA and Duncan’s multiple range test were performed using SPSS software (version 27.0) to test for variance and significant differences.

## Results and discussion

### Strain screening and identification

The results of 16S rDNA sequence of strain T22.7.1^T^ compared in Ezbiocloud database showed that strain T22.7.1^T^ was similar to *Rhodococcus ruber* DSM 43338^T^ (98.96%), *Rhodococcus electrodiphilus* JC435^T^ (98.93%) and *Rhodococcus aetherivorans* 10bc312^T^ (98.90%) had the highest similarity, while the rest of the strains of the genus *Rhodococcus* had similarities below 97.80%. The NCBI database comparisons showed similar results, with strain T22.7.1^T^ being similar to *R. ruber* DSM 43338^T^, *R. electrodiphilus* JC435^T^, and *R. aetherivorans* 10bc312^T^ with similarities of 98.97, 98.81, and 98.83%, respectively, and additional comparisons found that strain T22.7.1^T^ was similar to the most recently reported (January 2024) *R. indonesiensis* CSLK01-03^T^ with the highest similarity of 99.79%.

**Figure 1:**
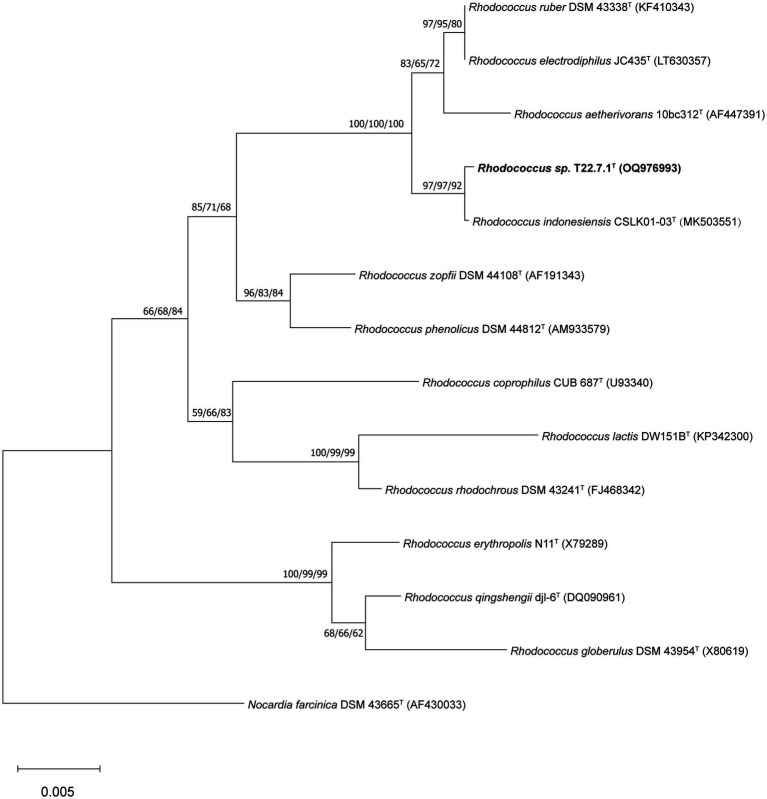
Phylogenetic tree based on 16S rDNA sequences showing the relationship of strain T22.7.1^T^ to the most closely related members of the genus *Rhodococcus*. The tree was constructed using the NJ method and the phylogenetic tree was reconstructed using the ML and MP methods, with the number before each branch node representing the bootstrap value and the percentage of bootstrap involved in the NJ/ML/MP analysis, and the numbers below 50% percentage of bootstrap were omitted. *Nocardia farcinica* DSM 43665^T^ was defined as an outgroup of the tree and used as the root. The GenBank accession number for the 16S rDNA sequence of each strain is listed in parentheses. The scale bar indicates 0.005 substitutions per nucleotide position.

**Figure 2:**
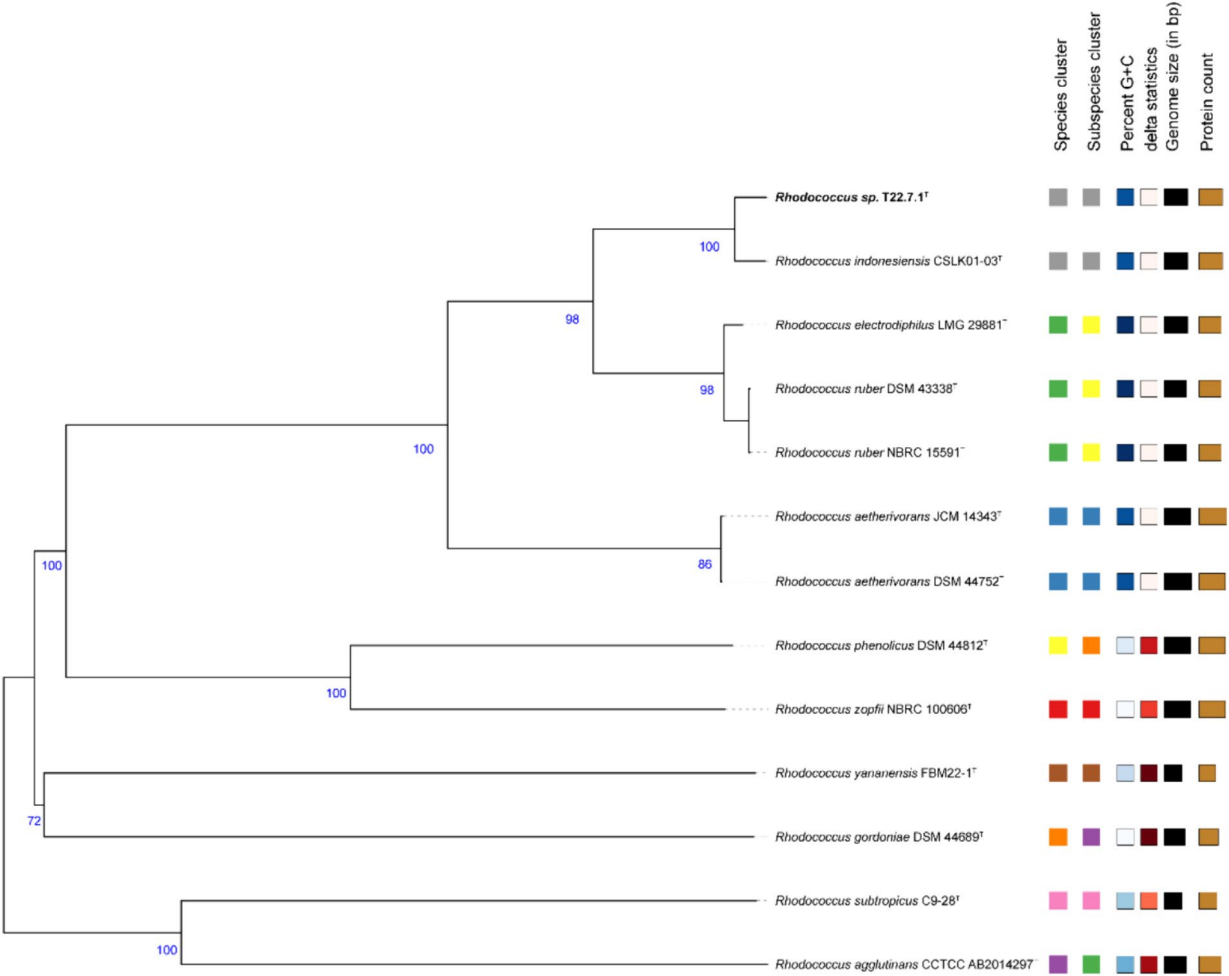
Phylogenetic tree based on whole-genome sequences. This tree is inferred with FastME 2.1.6.1 in the TYGS website from genome blast distance phylogeny (GBDP) approach. Branch length is scaled by GBDP distance formula d5. The number of branches above is 100 replicates of GBDP pseudo-guidance support value >60%, and the average branch support is 90.9%. The tree was rooted at the midpoint.

The phylogenetic tree constructed using the aligned 16S rDNA sequences (full length 1,473 bp) showed that strain T22.7.1^T^ was most closely related to the strain *R. indonesiensis* CSLK01-03^T^, as well as to *R. ruber* DSM 43338^T^, *R. electrodiphilus* JC435^T^, and *R. aetherivorans* 10bc312^T^. *R. indonesiensis* CSLK01-03^T^ clustered as a branch of the evolutionary tree (Figure 1), consistent with the phylogenetic tree constructed based on the whole-genome sequence of the strain (Figure 2), verifying that the strain is a member of the genus *Rhodococcus*.

And based on the whole genome sequence comparison analysis, it was found that the genome-to-genome distance between strain T22.7.1^T^ and strains *R. indonesiensis* CSLK01-03^T^, *R. ruber* DSM 43338^T^, *R. electrodiphilus* JC435^T^, and *R. aetherivorans* 10bc312^T^ were 0.0101, 0.0489, 0.0493 and 0.0882, respectively, from which the evolutionary relationship between the strains and their close relatives could be inferred.

**Figure 3:**
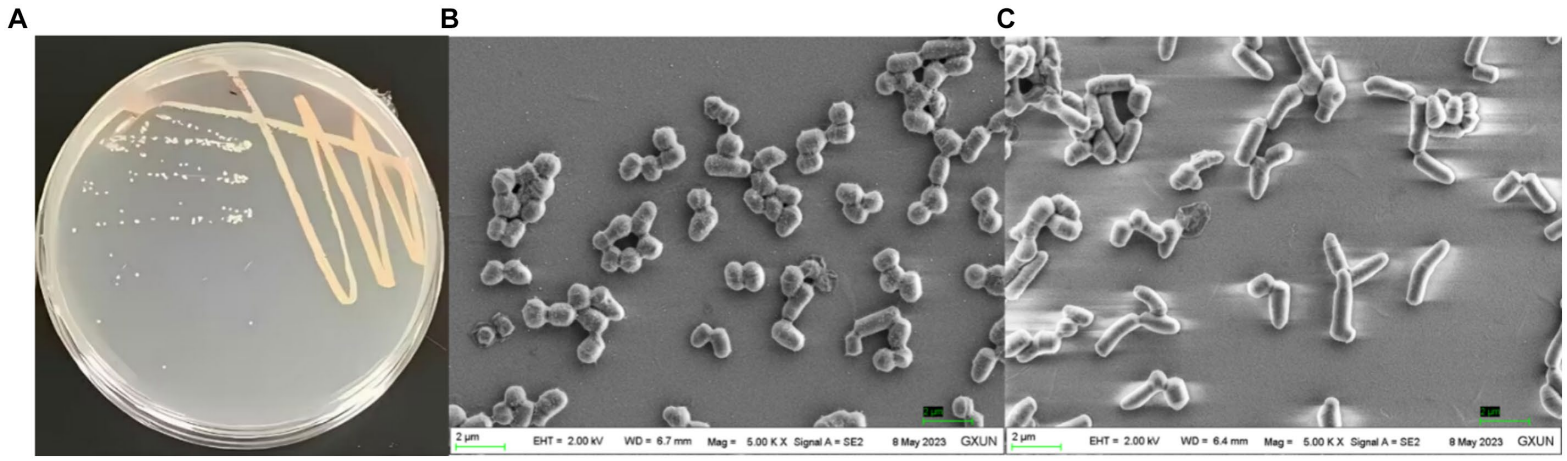
Culture morphology **(A)** and cell morphology **(B,C)** under scanning electron microscopy of the newly isolated strain T22.7.1^T^. **(A)** shows the strain cultured on LB medium for 2 days when the color of the strain started to change from light yellow-white to orange-red; **(B)** shows the cells of the strain appearing in the shape of a sphere with a magnification of 5,000; **(C)** shows the cells of the strain appearing in the shape of a rod with a magnification of 5,000.

The culture morphology of strain T22.7.1^T^ on screening plate media and LB agar media is shown in Figure 3A and Supplementary Figure S1A. The purified strain T22.7.1^T^ showed a single colony of nondiffuse growth on the screening medium, with a light yellow–white color. Prolonged incubation led to the deacetylation of p-nitroacetanilide in the light-yellow medium, resulting in a brighter golden yellow color (Supplementary Figure S1A). This indicates that the strain can produce chitin deacetylase, making it a target strain for studying chitin/CTO deacetylation (Liping, 2008). Compared with screening media supplemented with colloidal chitin as the sole carbon source, LB media (after 2 days of incubation the growth was denser, and the strain began to shift from a light yellowish-white color to an orange-red coloration, whereas it was slow-growing and did not produce any red pigmentation on the screening medium) provided more favourable growth conditions for strain T22.7.1^T^, as evidenced by its faster growth rate on LB media. Morphological characteristics of strain T22.7.1^T^ on other media are recorded in Supplementary Table S1.

The cell morphology of strain T22.7.1^T^ was short rod-shaped (1.8–3.2 μm) to globular (1.2–1.6 μm) (Figures 3B,C), a characterization that is consistent with that of its close species relatives, strains *R. indonesiensis* CSLK01-03^T^, *R. electrodiphilus* JC435^T^, *R. ruber* DSM 43338^T^, and *R. aetherivorans* 10bc312^T^, but the cell size of strain T22.7.1^T^ is slightly longer (Ramaprasad et al., 2018; Kusuma et al., n.d.). Additionally, at 100,000× magnification, two distinct rings of annular protrusions were observed at the ends of the cells, along with terminal bulbous protrusions forming a cap-like structure (Supplementary Figure S1B), a feature not previously reported for closely related species of strain T22.7.1^T^. The cell morphology of strain T22.7.1^T^ exhibited two or more cells side-by-side or in tandem, differing markedly from that of its close relatives: *R. electrodiphilus* JC435^T^, *R. ruber* DSM 43338^T^, and *R. aetherivorans* 10bc312^T^; strain *R. electrodiphilus* JC435^T^ cells arranged in rows of 4–5 cells; *R. ruber* KCTC 9806^T^ cells mostly forming tetrads; and *R. aetherivorans* JCM 14343^T^ cells mostly bundled (Ramaprasad et al., 2018). Additionally, strain T22.7.1^T^ proved to be an aerobic, Gram-stain-positive, nonmotile strain that was able to tolerate up to 12% (w/v) NaCl (optimal concentration 1% (w/v)), grow in a pH range of 5–9 (optimal pH 8), and thrive at temperatures ranging from 10 to 45°C (optimum 35°C). More morphological and cultural characteristics as well as physiological and biochemical characteristics are detailed in Table 1 and Supplementary Table S2. The morphological, biochemical, and physiological characteristics of strain T22.7.1^T^ conform to the typical characteristics of *Rhodococcus* strains (Parte et al., 2012; Lee et al., 2020; Sun et al., 2023).

**Table 1:** Differential characteristics between strain T22.7.1^T^ and its closely species strains.

Characteristics	1	2	3	4	5
Isolation source	Mudflat sediment	Hot spring sediment	Marine coral reef	Sediment	Petrochemical biotreater sludge
Colony colour	Orange red	Reddish orange	Dark red	Pale orange	Pinkish
Temperature range for growth (°C)	10–45	10–39	20–40	10–40	10–40
NaCl range for growth (%, w/v)	0–12	0–10	0–13	0–12	0–12
pH tolerance for growth	5.0–9.0	6.5–8.0	6.5–11.0	5.5–8.5	6.0–8.5
Hydrolysis of:					
Casein	−	−	+	−	+
Starch	−	−	+	+	+
Tween 20	−	−	−	+	−
Tween 60	−	−	+	+	−
Tween 80	−	−	+	+	−
Chitin	−	+	+	−	−
Urea	−	−	−	+	−
Xylan	−	+	+	−	+
Growth on sole carbon sources:					
*myo*-Inositol	+	+	+	−	−
Sodium Butyrate	−	−	+	+	−
Sodium Gluconate	+	+	−	+	−
_D_-mannitol	+	−	+	−	+
_D_-sorbitol	+	+	+	+	−
Maltose	+	+	−	+	+
N-Acetyl glucosamine	+	−	+	−	+
Cellobiose	+	+	+	−	−
_D_-galactose	+	−	+	−	+
Cellulose	−	−	+	−	−
Sucrose	+	−	+	−	+
_L_-rhamnose	+	−	+	−	−
Phospholipids^†^	DPG, PME, NPG, PG, PI, PLs, GLs, PIM, PIDM	DPG, PE, PME, PI, PIM, GLs, PLs	DPG, PE, PI, PIM, PLs, ULs, AL	DPG, PE, PI, PIM, PLs	DPG, PE, PI, PIM, PL
Major fatty acids	C_16:1_ ω6c/ω7c, C_16:0_, C_18:0_ 10-methyl- TBSA	C_16:0_, C_18:1_ ω9c, C_18:0_ 10-methyl-TBSA, C_18:2_ ω6/anteiso-C_18:0_ 9c	C_16:0_, C_17:1_ iso I/anteiso B, C_18:1_ ω9c, C_18:0_ 10-methyl-TBSA	C_16:1_ ω6c/ω7c, C_16:0_, C_18:1_ ω9c, C_18:0_ 10-methyl- TBSA	ND
Major menaquinone	MK-7(H2), MK-8, MK-8(H2)	MK-8, MK-8(H2)	MK-8(H2), MK-7(H2), MK-9(H2)	ND	ND

^†^DPG, diphosphatidylglycerol; PME, phosphatidylmethylethanolamine; PC, phosphatidylcholine; PE, phosphatidylethanolamine; PI, phosphatidylinositol; LPG, lyso-phospatidyl glycerol; PGL, phosphatidylglycerides; PG, phosphatidylglycerol; PS, phosphatidylserine; PIM, phosphatidylinositol mannosides; PIDM, phosphatidylinositol dimannosides; NPG, phospholipids of unknown structure containing glucosamine; PLs, unknown phospholipids; GLs, unidentified glycolipid; ULs, unidentified lipids; ALs, unidentified aminolipids. Strains: 1, T22.7.1^T^; 2, R. indonesiensis CSLK01–03^T^ [*data from Kusuma et al., n.d.]; 3, R. electrodiphilus JC435^T^ [*data from Ramaprasad et al., 2018]; 4, R. ruber DSM 43338^T^ [*data from Ramaprasad et al., 2018]; 5, R. aetherivorans 10bc312^T^ [*data from Ramaprasad et al. (2018)]. −, Negative; +, positive; w, weakly positive; ND, not determined.

### Chemical taxonomic characterization

The whole-cell sugars of strain T22.7.1^T^ were identified as glucose and galactose. The characteristic diamino acid, meso-diaminoheptanedioic acid, was detected in the cell wall hydrolysate of the new isolate. The major fatty acid fractions (>10%) of strain T22.7.1^T^ were C_16:1_ ω6c/C_16:1_ ω7c (10.76%), C_16:0_ (26.81%), and C_18:0_ 10-methyl-tuberculostearic acid (TBSA) (14.97%), and the detailed distribution of fatty acid content is shown in Supplementary Table S3. In the strain T22.7.1^T^, 12 lipids were detected, which were diphosphatidylglycerol, phosphatidylmethylethanolamine, phosphatidylinositol, phosphatidylglycerides phosphatidylinositol mannosides, phosphatidylinositol dimannosides, two unknown glycolipids, two phospholipids of unknown structure containing glucosamine and two unknown lipids (Supplementary Figure S2). The predominant respiratory quinone type (>10%) of strain T22.7.1^T^ was MK-8(H2) (96.3%), with small amounts of MK-7(H2) (2.3%) and MK-8 (1.4%) also detected. All the chemotaxonomic features of strain T22.7.1^T^ were consistent with the genus *Rhodococcus* (Parte et al., 2012; Lee et al., 2020; Zhang et al., 2021; Sun et al., 2023). Comparison of chemical taxonomic characters between strain T22.7.1^T^ and its close relatives is shown in Table 1.

### Comparative genomic analysis

**Figure 4:**
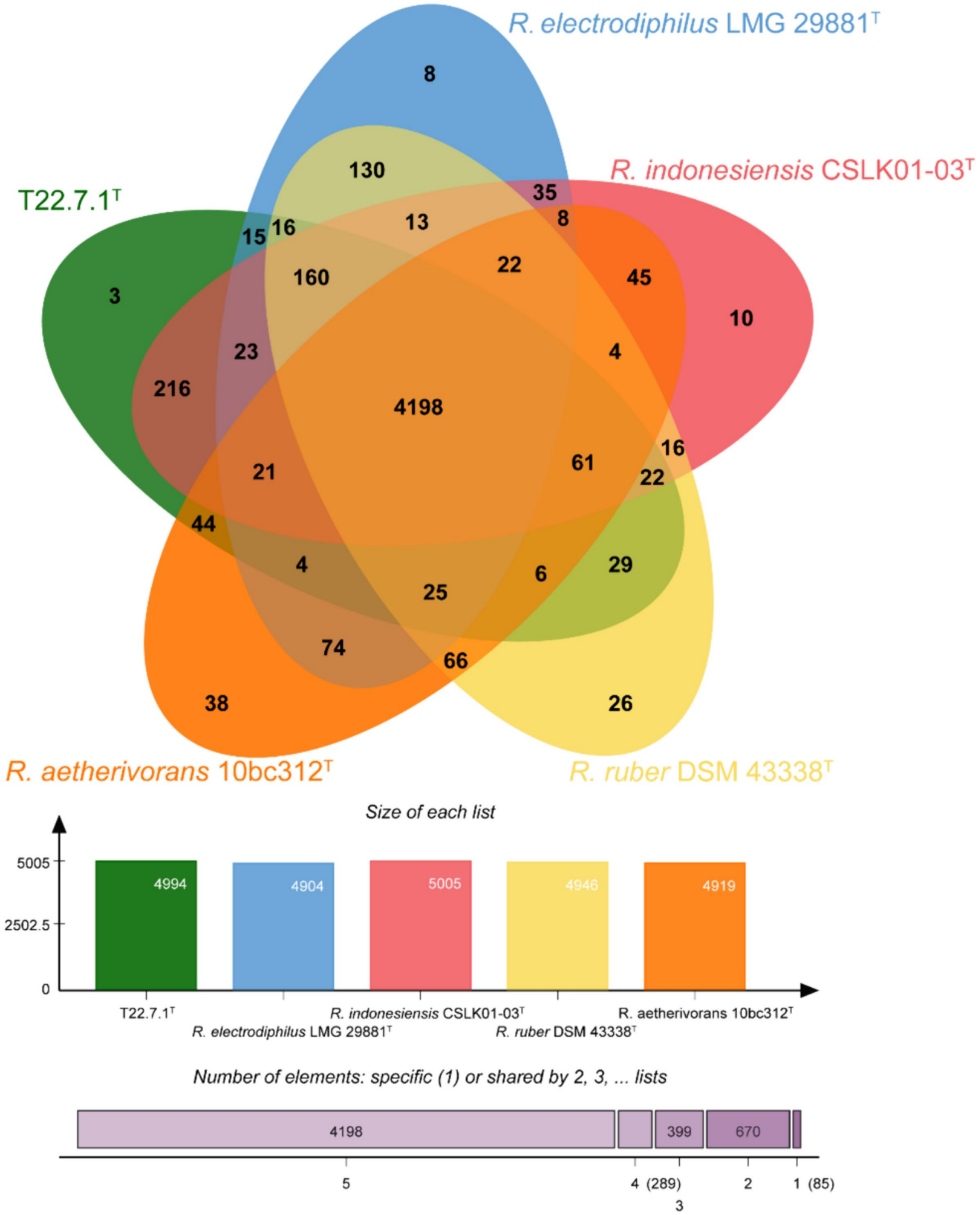
Venn diagram representing the core orthologs and unique genes for strain T22.7.1^T^ and closely related type strains.

The core and specific genes of strain T22.7.1^T^ and the strains most relevant to its development were determined using OrthoVenn3 analysis. A total of 4,198 core gene clusters were identified in the five strains (Figure 4), of which six genes were specific to T22.7.1^T^, which encodes functional small molecules such as deconjugating enzymes, hydrogen sulfide hydrolysis, and nitrogen-containing compound hydrolysis activities. A comparison of the number of homologous genes revealed that T22.7.1^T^ had the highest similarity 90.93% (5,102/5611) with *R. indonesiensis* CSLK01-03^T^. However, *R. electrodiphilus* JC435^T^ (84.70%), *R. ruber* DSM 43338^T^ (84.42%), *R. aetherivorans* 10bc312^T^ (74.49%) showed low sequence similarity. In addition, the ANI and dDDH values based on whole-genome sequences showed that strains T22.7.1^T^ and *R. indonesiensis* CSLK01-03^T^ had the highest similarity (98.90/91.90), exceeding the critical values for the delineation of the same species (ANI value >95%, dDDH value >70%), whereas strains T22.7.1^T^ and *R. aetherivorans* 10bc312^T^ (74.49%) had lower sequence similarity (Table 2). Although strain T22.7.1^T^ had ANI and dDDH values lower than the critical values for three other closely related strains, strain T22.7.1^T^ can be recognized as another type of strain under the species classification of *R. indonesiensis* (Goris et al., 2007; Richter and Rossello-Mora, 2009). In this study, we found that the ANI and dDDH values (98.76/92.50) between *R. ruber* DSM 43338^T^ and *R. electrodiphilus* JC435^T^ also exceeded the critical values for species classification, validating recent findings by Kusuma et al. to recognize *R. ruber* DSM 43338^T^ and *R. electrodiphilus* JC435^T^ as the same species (Kusuma et al., n.d.). These results help to isolate strain T22.7.1^T^ from the closely related type strain and support the determination of its taxonomic status.

**Table 2:** ANI and dDDH values found between strain T22.7.1^T^ and its closest related type strains.

ANI/dDDH (%)	T22.7.1^T^	*R. indonesiensis* CSLK01-03^T^	*R. ruber* DSM 43338^T^	*R. electrodiphilus* JC435^T^	*R. aetherivorans* 10bc312^T^
T22.7.1^T^	100/100				
*R. indonesiensis* CSLK01-03^T^	98.90/91.90	100/100			
*R. ruber* DSM 43338^T^	94.90/61.60	94.85/61.40	100/100		
*R. electrodiphilus* JC435^T^	94.69/61.40	94.67/61.30	98.76/92.50	100/100	
*R. aetherivorans* 10bc312^T^	89.97/43.60	89.95/43.60	90.34/44.90	90.14/44.60	100/100

ANI value above cutoff (>95%) is displayed in green, and dDDH value above cutoff (>70%) is displayed in blue when they are compared in different strains.

Genome characterization, annotation and functional prediction:

The draft genome of strain T22.7.1^T^ consisted of 112 contigs with a total genome size of 5.53 Mbp and a G + C content of 70.17 mol%. These data are consistent with the characterization of draft genome sizes ranging from 3.9 to 10.4 Mbp and DNA G + C contents ranging from 61.8 to 70.7 mol% in the genus *Rhodococcus* (Nouioui et al., 2018; Zhang et al., 2021; Kusuma et al., n.d.). Based on the comparative analysis of genome size and G + C content, strain T22.7.1^T^ had a slightly larger genome (5.48 Mbp) and higher G + C content than *R. indonesiensis* CSLK01-03^T^ (Supplementary Table S5), indicating that strain T22.7.1^T^ is different from CSLK01-03^T^. The number of protein-coding genes and RNA genes was 5,092 and 67, respectively, and more features of strain T22.7.1^T^ genome annotation, such as comparison of annotation results from databases such as NR, Swiss-Prot, Pfam, eggNOG, GO and KEGG, are listed in Supplementary Table S4.

Secondary metabolites are generally controlled by multiple genes, which usually exist in clusters in the genome and encode complex enzymes with multiple functions. These clusters are the secondary metabolite biosynthesis-related gene clusters (smBGCs). The smBGCs of strain T22.7.1^T^ and its close relatives were predicted by antiSMASH. A total of 24 putative smBGCs were detected in strain T22.7.1^T^. The identified smBGCs showed homology to 14 gene clusters with known metabolites such as ε-poly-L-lysine, ectoine, heterobactin A/heterobactin S2, and isorenieratene. In addition, strain T22.7.1^T^ contains a unique gene cluster (Liu et al., 2014), the stenothricin synthesis gene cluster. The smBGCs of *R. indonesiensis* CSLK01-03^T^, *R. ruber* DSM 43338^T^, *R. electrodiphilus* JC435^T^, and *R. aetherivorans* 10bc312^T^ were also analysed, and the type and number of their encoded gene clusters differed from those of strain T22.7.1^T^.

Comparing the smBGCs of T22.7.1^T^ and *R. indonesiensis* CSLK01-03^T^, in addition to stenothricin, there were also cinnapeptin, coelichelin, and SF2575 that were present in the former but absent in the latter; on the other hand, the smBGCs involved in the synthesis of ohmyungsamycin A/B, the rhizomide A/B/C, lymphostin/neolymphostinol B/lymphostinol/neolymphostin B, madurastatin A1/A2/E1/F/G1, and corynecin I/II/III are present in *R. indonesiensis* CSLK01-03^T^, but not in strain T22.7.1^T^ (Supplementary Table S6). These results further illustrate the divergence in metabolic potential between strain T22.7.1^T^, a new species of *R. indonesiensis*, and its developmentally most relevant strain.

Carbohydrate-active enzymes (CAZymes) are a large group of enzymes with functions related to degrading, modifying, and generating glycosidic bonds (Yin et al., 2022; Lopez-Sanchez et al., 2024). In-depth studies on CAZymes are important for understanding carbohydrate metabolism in microorganisms. Carbohydrate esterases (CEs), carbohydrate-binding modules (CBMs), auxiliary oxidoreductases (AAs), and six other protein families were annotated in the genomes of strain T22.7.1^T^ and its close relatives by CAZy. Strain T22.7.1^T^ possesses genes for synthesizing the above six classes of CAZymes, whereas polysaccharide lyase family 12/subfamily 3 is present only in strains T22.7.1^T^ and *R. ruber* DSM 43338^T^ but not in the other three reference strains (Supplementary Table S7). Reports on the functional activity of the PL 12_3 subfamily are lacking, but heparinase II/III of the PL 12 subfamily (EC 4.2.2.8) is widely used as a CAZyme for the production of clinically and therapeutically relevant bioactive heparin oligosaccharides (Balasubramaniam et al., 2018). Finally, CDA, which is of interest in this study, also belongs to one of the CAZyme and is categorized in the CE4 family, whose members are currently chitin oligosaccharide deacetylase (EC 3.1.1.-), acetylesterase (EC 3.1.1.6), acetylxylan esterase (EC 3.1.1.72), LPS deacetylase (EC 3.5.1.-), Poly-β-1,6-N-acetylglucosamine deacetylase (EC 3.5.1.-), Peptidoglycan N-acetylglucosamine deacetylase (EC 3.5.1.-). acetylglucosamine deacetylase (EC 3.5.1.104), and Chitin deacetylase (EC 3.5.1.41) (Araki and Ito, 1974; Andres et al., 2014; Nakamura et al., 2017). Comparative analysis revealed that strains T22.7.1^T^, *R. indonesiensis* CSLK01-03^T^ and *R. aetherivorans* 10bc312^T^ all have only one gene encoding a gene that synthesizes functional proteins of the CE 4 family, while *R. ruber* DSM 43338^T^ and *R. electrodiphilus* JC435^T^ are absent, reflecting on the one hand the functional potential of members of the genus *Rhodococcus* to produce CDA, and on the other hand suggesting that not all *Rhodococcus* strains are capable of chitin deacetylation.

Taken together, the morphological, physiological, biochemical characteristics, chemical taxonomic data, and comparative genomic analysis results of strain T22.7.1^T^ suggest that it is another member of the genus *Rhodococcus* that is most closely related to *R. indonesiensis* CSLK01-03^T^. However, the polyphasic taxonomy results indicate that they are different, and therefore, strain T22.7.1^T^ is proposed as another type strain representing the *R. indonesiensis* species.

### Optimization of fermentation conditions

During the initial screening of the strains, the activity of the CDA enzyme produced was tested under basal fermentation conditions. The results showed that the enzyme activity of the CDA enzyme produced by the strains under unoptimized conditions for 72 h of fermentation was 8.0 ± 2.2 U/mL. The fermentation broth was further centrifuged, and the enzyme activity in the precipitate and the supernatant was tested. The enzyme activity of the fermentation broth was taken as 100%, and the enzyme activity in the precipitate reached 90%, indicating that strain T22.7.1 produces CDA (*Ri*CDA), which is located in the bacterium and is hardly secreted outwards. Thus, all subsequent optimizations were tested with the fermentation broth to determine the enzyme activity directly after fermentation. The liquid fermentation medium and culture conditions of strain T22.7.1 were optimized by one-factor optimization and orthogonal design, aiming to increase the enzyme activity of CDA produced by the strain per unit volume, providing sufficient CDA enzyme for subsequent enzyme purification and enzymatic characterization studies. After one-way optimization, the optimal carbon source of the medium was determined to be 1.5% (w/v) sucrose, the optimal nitrogen source was 3.5% (w/v) yeast extract, the optimal inorganic salt was 0.01% (w/v) magnesium sulfate, and the optimal inducer was 0.06% (w/v) p-nitroacetanilide. Additionally, the optimized medium had an initial pH of 8, a loading volume of 30% (v/v), an inoculum volume of 4% (v/v), a fermentation temperature of 28°C, and a fermentation time of 2 days (Table 3; Supplementary Figures S3, S4). The highest enzyme activity after one-factor optimization was 287.02 U/mL, which was 35.88 times greater than that before optimization (8.0 U/mL). In contrast, the enzyme activity of *R. erythropolis* HG05, optimized by Plackett–Burman and central composite design (238.89 U/mL), was still lower than that of *R. indonesiensis* T22.7.1 (Sun et al., 2014), suggesting that the T22.7.1 strain has potential application in high CDA production.

**Table 3:** Optimization of fermentation conditions for CDA production of strain T22.7.1.

Fermentation conditions	Optimization condition	Enzyme activity (U/mL)
Carbon source (%, w/v)	Sucrose (1.5)	7.44 ± 0.24
Nitrogen source (%, w/v)	yeast extract (3.5)	44.44 ± 4.67
Inorganic salt (%, w/v)	Mg_2_SO_4_ (0.01)	10.25 ± 1.70
Inducer (%, w/v)	p-nitroacetanilide (0.06)	287.02 ± 23.19
Initial pH	8	104.84 ± 18.34
Medium volume (ml/150 mL)	50	56.81 ± 8.04
Inoculum size (%, v/v)	4	66.88 ± 1.96
Fermentation temperature (°C)	28	48.11 ± 7.94
Fermentation time (days)	2	51.44 ± 6.92

### Enzyme purification, characterization and production analysis

**Figure 5:**
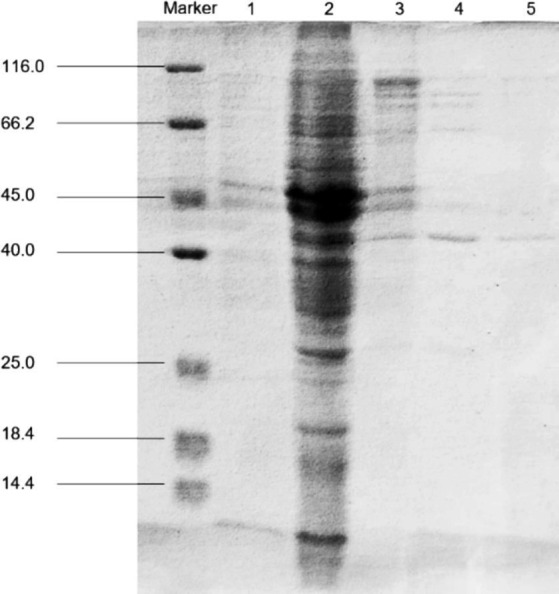
SDS-PAGE for purification analysis of CDA enzyme produced by strain T22.7.1. Marker, protein molecular weight markers; line 1, crude extract; line 2, crude CDA concentrated 10 times after ammonium sulfate precipitation; line 3, purified by Q Sepharose; line 4, purified by molecular sieve; line 5, secondary purification by molecular sieve.

The examination of fermentation products for the presence of CDA indicated that *Ri*CDA was intracellular. To extract the crude CDA, ultrasonic disruption of the fermented bacteria was employed, yielding a crude enzyme solution. This solution underwent a series of purification steps—75% saturation ammonium sulfate precipitation, anion exchange chromatography, and gel filtration chromatography—to achieve basic purification. From 100 mL of crude enzyme solution, 1 mL of purified CDA was obtained, representing a purification factor of 31.83-fold and a specific activity of 1200.33 U/mg, as shown in Table 4. The purification process was further assessed using SDS–PAGE, the results of which are depicted in Figure 5. The crude enzyme solution contained numerous heterogeneous proteins, which were less apparent in Line 1. After ammonium sulfate precipitation, which removed some heterogeneous proteins and concentrated the sample by 10-fold (line 2), heterogeneous proteins were still prevalent. Subsequently, a single band of pure protein was isolated through ion exchange and gel filtration chromatography. Protein electrophoresis revealed that the CDA produced by fermentation of strain T22.7.1 had an apparent molecular weight of approximately 42 kDa and was present as nearly a single component. This molecular weight is inconsistent with theoretical predictions for CE4 family proteins (consisting of 281 amino acids and a relative molecular weight of 29 kDa) annotated in the genome. Previous studies suggest that most CDAs, being glycoproteins, typically range from 40 to 80 kDa and often appear in multiple isoforms (Deising and Siegrist, 1995; Tsigos and Bouriotis, 1995; Kim et al., 2008).

**Table 4:** Purification results of *Ri*CDA by ammonium sulfate precipitation, ultrafiltration, anion exchange chromatography, and gel filtration chromatography.

Purification steps	Volume (mL)	Protein conc. (mg/mL)	Enzyme activity (U/mL)	Specific activity (U/mg)	Activity yield (%)	Purification fold
Crude extract	100	6.42	242.10	37.71	100	1
Ammonium sulfate	21	9.82	936.84	95.40	81.26	2.53
Ultrafilter	17	3.27	575.91	176.12	40.44	4.67
Q Sepharose	4	0.67	383.32	572.12	6.33	15.17
Superdex 75	1	0.24	288.08	1200.33	1.19	31.83

### Enzymatic property tests

**Figure 6:**
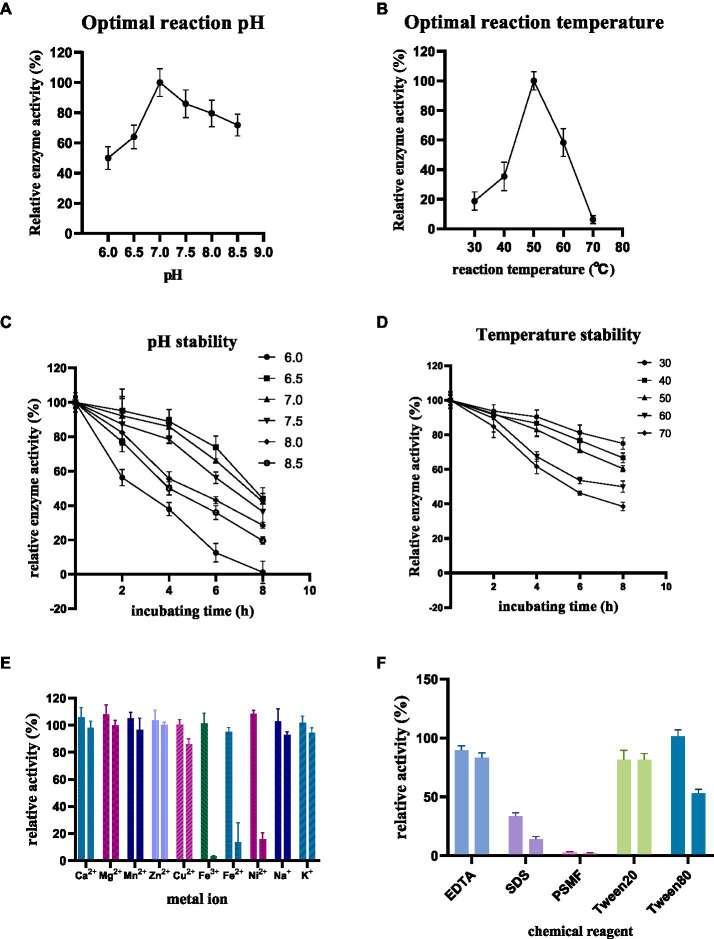
Enzymatic properties of purified *Ri*CDA **(A,B)**, Effect of pH and temperature on enzyme activity of *Ri*CDA **(C,D)**, effect of incubation at different pH and temperature for 0–8 h on enzyme activity of *Ri*CDA **(E,F)**, effect of metal ions and chemical reagents on enzyme activity of *Ri*CDA, each color represents a metal ion or chemical reagent, and the final concentration of ions in the reaction system is 1 mM on the left and 5 mM on the right. The error bar represents Mean ± SD.

The enzyme activity of purified *Ri*CDA was assessed under various conditions, including pH, temperature, incubation time, and reaction conditions in the presence of metal ions and chemical reagents. This investigation aimed to identify the factors that influence enzymatic deacetylation and their impact on enzyme activity. The findings revealed that the optimal pH for *Ri*CDA was 7.0, with the enzyme activity remaining above 50% of its maximum level within the range of 6.0–8.5 (Figure 6A). *Ri*CDA exhibited the highest enzyme activity at 50°C, and any deviation of 10°C above or below this temperature led to a rapid decrease in activity. Furthermore, enzyme activity was almost completely lost at temperatures of 70°C or higher due to enzyme inactivation (Figure 6B). Figures 6C,D depict the enzyme activity of *Ri*CDA under various pH buffer systems and temperatures. *Ri*CDA showed the greatest stability at pH 6.5, followed by pH 7, as evidenced by its similar performance. Even after incubation at 50°C for 8 h, the relative enzyme activity remained at approximately 50%. Notably, the enzyme activity of *Ri*CDA incubated at 50°C for 8 h did not differ significantly. Moreover, in the temperature stability test showed that at low temperature (<50°C), the enzyme activity was lost slowly decreased gradually during prolonged incubation, and the lower the temperature, the slower the at low temperatures (<50°C). After 8 h of incubation at these temperatures, the enzyme activity was maintained at approximately 80%. These data results indicate that *Ri*CDA has high exhibits remarkable stability when stored at pH 7.0 and temperatures below 30°C, which provides experience. Such insights provide valuable guidance for the subsequent efficient preservation of the *Ri*CDA enzyme.

Metals serve as cofactors in enzymatic catalysis and can enhance the activity of specific enzymes. To determine the metal ions essential for catalysis, the impact of various metal ions and EDTA on chitinase activity was investigated. The findings demonstrated that at low concentrations (1 mM), Ca^2+^, Mg^2+^, Mn^2+^, Zn^2+^, Fe^3+^, Ni^2+^, and Na^+^ ions stimulated the enzyme activity of *Ri*CDA. Notably, Mg^2+^ and Ni^2+^ exhibited a more substantial promotional effect (Figure 6E), increasing the relative enzyme activity by 8.1 and 8.4%, respectively. However, compared to CDA strains from different origins (the metal ions that had a promoted metal ions up to more than doubling the relative enzyme activity), the promotion observed was less pronounced (Bai et al., 2020; Ding et al., 2021; Yang et al., 2022a). Conversely, high concentrations (5 mM) of Cu^2+^, Fe^3+^, Fe^2+^, and Ni^2+^ significantly inhibited the enzyme activity of *Ri*CDA, particularly Fe^3+^, which completely halted its activity (Figure 6E). Previous research has shown that the addition of Zn^2+^, Mg^2+^, Co^2+^, Mn^2+^, and Ba^2+^ to the reaction system of CDA enzymes from certain bacterial strains can enhance the reaction activity. Conversely, Cu^2+^ and Ni^2+^ can substantially obstruct enzyme activity. It should be noted that the effects of these metal ions are not absolute and may yield entirely opposite effects for some CDA strains (Bai et al., 2020; Chai et al., 2020; Yang et al., 2022a). Additionally, the introduction of chemical reagents such as EDTA, SDS, and PSMF considerably hindered the activity of *Ri*CDA (Figure 6F), regardless of the concentration. The inhibition of CDA activity by EDTA has also been documented in previous studies on CDA activity from various sources. This inhibition is attributed to the ability of EDTA, as a metal ion chelator, to chelate metal ions (e.g., Mg^2+^ and Ca^2+^ in solution), thus reducing the activity of metal-dependent proteins (Ding et al., 2021; Yin et al., 2022). SDS, an ionic surfactant, binds to proteins, leading to denaturation and precipitation. Conversely, the inhibition of *Ri*CDA activity by phenylmethylsulfonyl fluoride (PMSF) may be ascribed to the specific binding of PMSF, a serine protease inhibitor, to serine residues at the enzyme’s active site, resulting in enzymatic inhibition (Valenzuela et al., 2023; Rani et al., 2024). Furthermore, Tween 20 mildly inhibited *Ri*CDA activity, whereas at a low concentration (1 mM), Tween 80 promoted *Ri*CDA activity but significantly suppressed enzyme activity at a high concentration (5 mM) (Figure 6F). This can be attributed to the fact that Tween 80 acts as a surfactant, stabilizing proteins through interfacial competition at low concentrations. Conversely, at high concentrations, Tween 80 reduces the accessibility of the enzyme’s active site, leading to decreased enzyme-substrate affinity (Fahmy and El-Deeb, 2023).

The substrate preference of *Ri*CDA for various substrates, including chitin, chitosan, and COS, was determined using HPLC to analyse the change in acetic acid content in the enzymatic deacetylation products. Among these substrates, NAG, CTO, and CSO are all water soluble. The results indicated that *Ri*CDA exhibited the highest enzyme activity towards CTO, followed by NAG, and the lowest activity towards CSO, suggesting a strong affinity of the CDA enzyme for acetylated CTO and NAG. Furthermore, the different durations of the enzymatic reaction of CTO demonstrated that the enzyme activity of *Ri*CDA decreased as the reaction time increased. However, even so, compared to that of the insoluble substrates chitin and chitosan, the enzyme activity of *Ri*CDA was highest for CTO when the reaction time was the same (3 h) (Table 5). Additionally, the deacetylation activity of *Ri*CDA was greater for chitin than for chitosan, possibly due to the higher acetylation level of chitin. In conclusion, *Ri*CDA exhibited deacetylation activity for all the mentioned substrates but displayed higher activity for substrates with a higher degree of N-acetylation and much higher activity for CTO than for CSO.

**Table 5:** Enzyme activity of *Ri*CDA for different substrates. -, presents no test.

Enzyme activity(U/mg)			
Substrate	Enzymatic reaction time (h)		
	1	3	6
10 mg/mL NAG	52.42 ± 4.48	–	–
20 mg/mL CTO	217.58 ± 12.33	91.12 ± 8.02	75.08 ± 10.23
20 mg/mL CSO	25.42 ± 2.31	–	–
50 mg/mL chitosan	–	2.92 ± 0.02	–
50 mg/mL chitin	–	27.75 ± 5.62	–

### Product characterization

**Figure 7:**
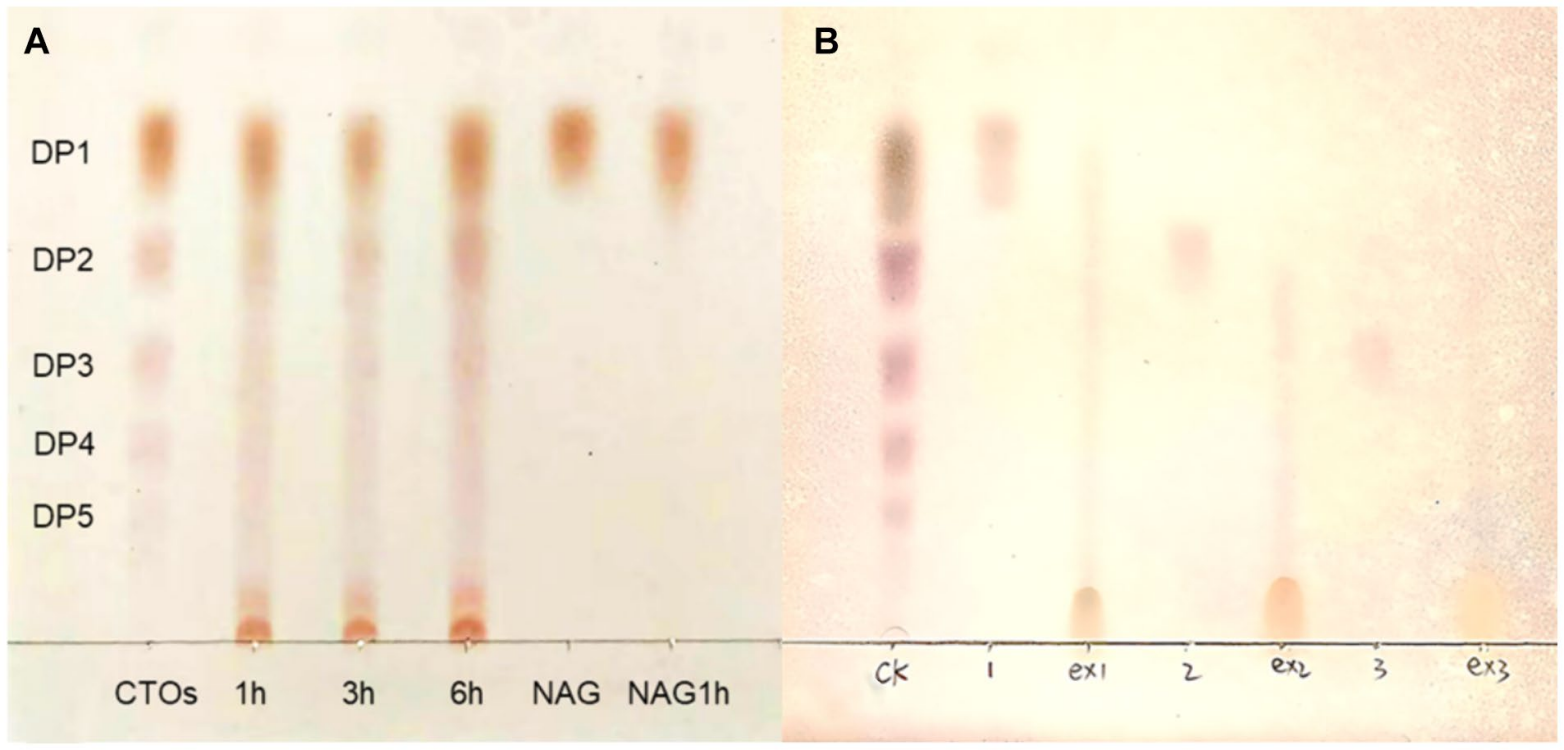
TLC results of chitin oligosaccharides deacetylated by *Ri*CDA. **(A)** CTOs was treated by *Ri*CDA for different times. **(B)** Comparison of oligosaccharides with 1–3 degree of polymerization before and after *Ri*CDA treatment.

The deacetylation products of CTO and NAG were analysed using TLC. When the acetyl group in oligosaccharides or monosaccharides is converted to an amino group, the polarity is increased, resulting in smaller retention factor values (Rf values) and shorter distances travelled by more polar substances (Rani et al., 2024). The results of the enzymatic products of *Ri*CDA are shown in Figure 7. The standard sample of CTO, which also includes NAG, was enzymatically deacetylated, resulting in a position lower than that of the standard CTO samples on TLC silica gel plates. This indicates that both the NAG and CTO of DP2-5 can be deacetylated by *Ri*CDA. In Figure 7A, spots very close to the spotting origin can be clearly observed in the 1 h, 3 h, and 6 h *Ri*CDA-treated groups. These spots correspond to completely deacetylated CSO, with the smallest Rf value due to the maximum polarity of CSO compared to that of CTO. Similar results were observed for *Ri*CDA-deacetylated NAG, chitobiose, and chitotriose (ex1, ex2, ex3), as shown in Figure 7B. Additionally, previous studies have shown that different sources of chitin deacetylases exhibit different patterns of enzymatic action on COS substrates (Naqvi et al., 2016; Grifoll-Romero et al., 2018; Bai et al., 2020; Bonin et al., 2020).

**Figure 8:**
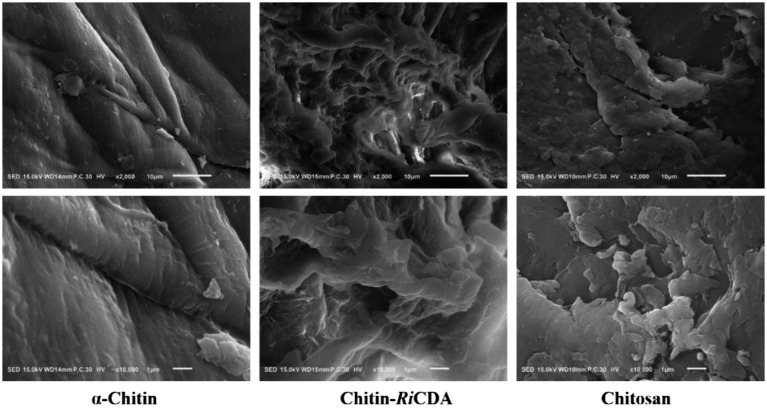
SEM images of untreated chitin, chitin treated by *Ri*CDA and chitosan with magnificent ×2,000 times (upper layer) and ×10,000 times (under layer).

Scanning electron microscopy (SEM) was used to characterize the α-chitin-, chitosan-, and *Ri*CDA-treated chitin (*Ri*CDA-chitin). The SEM results depicted in Figure 8 show that the untreated α-chitin exhibited a large, dense, layered structure in a lamellar arrangement, with a surface composed of closely packed crystalline microfibrillar structures that were dense, rough, and textured. However, in the case of *Ri*CDA-treated chitin, the original dense lamellar structure was profoundly damaged, resulting in the appearance of numerous irregular pits. The surface of *Ri*CDA-chitin exhibited holes and dense cracks, whereas its fibres became scattered and fuzzy, and the interface with the separated lamellae was less distinct. The particle state of *Ri*CDA-chitin resembled that of chitosan, which differed significantly from that of α-chitin (Yang et al., 2022a,b). As *Ri*CDA deacetylation results in a decrease in the acetyl group content on the glycan chains of chitosan, it leads to the disruption or weakening of the hydrogen bonding that maintains the intramolecular and intermolecular structural stability of the glycan chains, which results in the change of the microstructure of chitosan (Yang et al., 2023).

**Figure 9:**
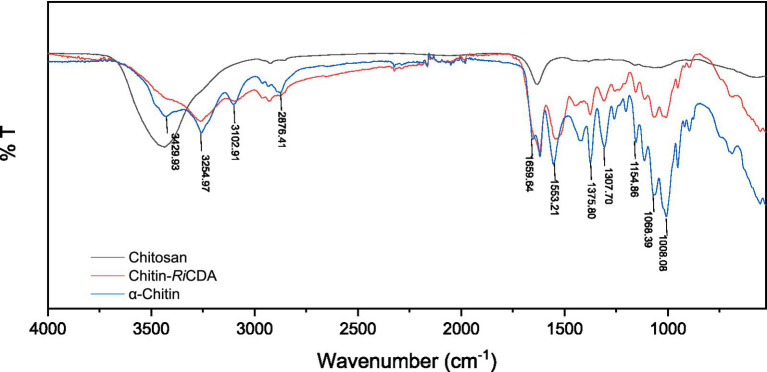
FT-IR spectra of chitin, chitosan, and *Ri*CDA treated chitin. Chitosan (black), *Ri*CDA treated chitin (red) and chitin (blue).

To identify the characteristic groups of *Ri*CDA-chitin, Fourier transform infrared spectroscopy (FT-IR) was conducted, and the results were compared with those of α-chitin and chitosan. The results, as presented in Figure 9, displayed a broader absorption peak at approximately 3,400 cm^−1^, resulting from the overlapping of -OH stretching vibrational peaks forming hydrogen bonding and -NH stretching vibrational peaks. Other significant peaks included the -CH stretching vibration at 2,928 cm^−1^, the amide I band at 1635 cm^−1^, and the amide II band at 1,558 cm^−1^. Additionally, the NH stretching vibration absorption peak overlapped and broadened multiple absorption peaks, including the -CH stretching vibration absorption peak at 2,928 cm^−1^, amide I spectral band at 1,635 cm^−1^, -NH deformation vibration absorption peak of amide II at 1,558 cm^−1^, 1,381 cm^−1^ for the -CH2 wobble absorption peak, amide III spectral band at 1,316 cm^−1^, asymmetric oxygen bridge telescopic vibrational absorption peak at 1,158 cm^−1^, and C-O telescopic vibrational absorption peaks at 1,073 and 1,027 cm^−1^ (Chai et al., 2020; Yang et al., 2022b, 2023). Notably, the -CH stretching peak at 2,928 cm^−1^ exhibited a shift to lower frequencies in correlation with the higher crystallinity of the chitin (Yang et al., 2022a). It is evident from the figure that the *Ri*CDA treatment resulted in relatively low crystallinity of the chitin. The DD was determined by the weakening of NH (3,269 cm^−1^), the C=O linkage of amide I (1,633 cm^−1^), the N-H linkage of amide II (1551–1,546 cm^−1^), and the band of amide III (1,378 cm^−1^) (Sixto-Berrocal et al., 2023). For analytical purposes, the amide I and III bands (1,655, 1,560, and 1,320 cm^−1^) were selected to calculate the deacetylation of chitin, chitosan, and *Ri*CDA-chitin due to the difficulty in determining the amide II band at high DDs. The results revealed that the DD of α-chitin was 36.43%, whereas that of *Ri*CDA-chitin was 83.70%, surpassing that of commercial chitosan (78.73%). Most of the identified CDAs have been reported to exhibit high activity against low molecular weight oligomers, whereas they are largely inactive against natural insoluble chitin (Kang et al., 2014; Naqvi et al., 2016). In this study, *Ri*CDA was found to be an effective chitinolytic agent to catalyze the deacetylation of chitin for the preparation of chitosan and to achieve value-added utilization of chitin bioresources.

## Conclusion

### Conclusions on the species identification of the new isolate

Based on the genomic, phylogenetic, and phylogenomic data, it can be inferred that strain T22.7.1^T^ is not only a member of the genus Rhodococcus but also a member of the *R. ruber* lineage. Through the use of phenotypic traits and the examination of dDDH and ANI values, it was possible to distinguish strain T22.7.1^T^ from its close relatives *R. aetherivorans* DSM 44752^T^, *R. electrodiphilus* LMG 29881^T^, and *R. ruber* DSM 43338^T^. However, differentiating it from *R. indonesiensis* CSLK01-03^T^ in terms of species status has proven challenging. Considering the taxonomic characteristics described earlier, the new isolate and *R. indonesiensis* CSLK01-03^T^ can be regarded as distinct type strains representing *R. indonesiensis*. Importantly, these associations are supported by ANI and dDDH similarities that exceed the thresholds used to classify closely related strains as the same species. Therefore, we propose that strain T22.7.1^T^ be designated as a post heterotypic synonym of *R. indonesiensis* (Kusuma et al., n.d.).

### Emended description of *Rhodococcus indonesiensis* (approved lists 2014)

Description is based on data from this and previous studies (Kusuma et al., n.d.).

Aerobic, Gram-stain-positive, non-motile actinomycetes, forming short rods (0.8–1.5 or 1.8–3.2 μm) and spherical elements (0.9–1.1 or 1.2–1.6 μm). The cell surface was smooth and showed cap-like projections at the ends, and the colony shifted from yellowish white to orange-red on ISP2 medium. They grew well on ISP series medium ISP1, ISP2, ISP3 and ISP6, were able to grow at temperatures ranging from 10 to 45°C, with an optimal growth temperature range of 28–35°C, were able to grow at pH ranging from 5.0–9.0, with an optimal pH around 7.0–8.0, were able to tolerate NaCl concentrations of 0–12% (w/v), and were able to grow at an optimal NaCl concentration of 1% (w/v). Catalase positive, oxidase negative, reduces nitrate, produces H_2_S, hydrolyzes picrin, allantoin, arbutin, gelatin, arginine and urea, produces lysine decarboxylase, does not produce phenylalanine deaminase. Degrades adenine, hypoxanthine, and uric acid but not casein, elastin, guanine, keratin, starch, casein tributyrate, xanthine, Tweens 20, 60, and 80. inositol, sodium gluconate, D-mannitol, D-sorbitol, maltose, NAG, cellulosic disaccharides, D-galactose, D-glucose, D-fructose, D-cotton seed sugars, glycerol, D-ribose, D-arabinose, trehalose, D-mannose, sucrose, L-rhamnose can be used as the sole carbon source, but arbutin, lecithin, cellulose, and sodium butyrate cannot. The characteristic diamino acid of the cell wall is meso-DAP. whole-cell hydrolysate sugars contain galactose, arabinose, or glucose. The major cellular fatty acids (>10%) were C_16:1_ ω6c/ω7c, C_16:0_, C_18:0_ 10-methyl-TBSA, the major methylnaphthoquinone (>10%) was MK-8 (H2) and the diagnostic polar lipids were diphosphatidylglycerol, phosphatidylmethylethanolamine, phosphatidylethanolamine, phosphatidylinositol, phosphatidylinositol mannosides, phosphatidylglycerol. Produces mycolic acid. The strain has a genome size of about 5.5 Mbp and a DNA G + C content of about 70.15 mol%.

Strain T22.7.1^T^ (=MCCC 1K08698^T^ = JCM36625^T^) was isolated from the rhizosphere of *Acanthus ebracteatus* in Beihai City, Guangxi Zhuang Autonomous Region, China. The GenBank accession number of the strain is JASKMB000000000.

### Fermentation optimization of strain T22.7.1, enzymatic properties and product characterization of *Ri*CDA

After optimizing the fermentation medium composition and culture conditions of strain T22.7.1, the enzyme activity per unit volume of the cultured strain reached 287.02 U/mL after 48 h. Additionally, the yield of CDA increased by 34.88 times compared to the non-optimized condition. This is solely the result of single-factor optimization. It is believed that further optimization of the fermentation process of strain T22.7.1 can significantly increase its CDA yield. The enzyme activity of CDA produced by the fermentation of T22.7.1 reached the highest reported level among strains of the same genus (Sun et al., 2014; Ma et al., 2020). *Ri*CDA was extracted and purified using a series of procedures, including cell wall disruption, ammonium sulphate precipitation, anion exchange chromatography, and gel filtration chromatography. The actual molecular weight of *Ri*CDA was approximately 42 kDa, which differed from the theoretical prediction of 29 kDa for CE4 family proteins noted in the genome. Previous studies have shown that *Ri*CDA is a glycoprotein with a protein subunit structure that differs from theoretical CDA molecules.

The purified *Ri*CDA exhibits excellent adaptability and stability to pH and temperature, and is capable of deacetylation activity within the pH range of 6.0–8.5 and temperature range of 30–70°C. Furthermore, it remains active even after continuous storage for 8 h within this range. *Ri*CDA exhibits the highest enzyme activity at pH 7.0 and 50°C. It can maintain approximately 70% enzyme activity for 8 h under these conditions, demonstrating its potential in the industrial deacetylation of chitin or chitin oligosaccharides for the preparation of chitosan and chitosan oligosaccharides. On the other hand, at low concentrations (1 mM), Ca^2+^, Mg^2+^, Mn^2+^, Zn^2+^, Ni^2+^, and other metal ions have a certain effect on the activity of *Ri*CDA, while high concentrations (5 mM) of Cu^2+^, Fe^3+^, and Fe^2+^ ions will inhibit its activity. Additionally, regardless of concentration, EDTA, SDS, PMSF, and Tween 20 inhibited the enzyme activity of *Ri*CDA, while Tween 80 helped to increase enzyme activity at low concentration and inhibited enzyme activity at high concentration. Regarding substrate adaptability, *Ri*CDA has a broad spectrum and can effectively deacetylate various substrates, such as chitin, chitosan, CTO, CSO, and NAG. Among these, CTO are the most suitable substrate, followed by NAG. *Ri*CDA also demonstrated deacetylation ability for chitin oligosaccharides with a polymerization degree of 1–5, further highlighting its potential applications.

Through the deacetylation of *Ri*CDA, natural chitin can be transformed into chitosan by removing the acetyl group on the sugar chain. This process reduces the structural stability of chitin and enhances its solubility, making it more conducive to degradation. In conclusion, strain T22.7.1 is a newly discovered strain with a relatively strong chitin deacetylation function. It has a controllable fermentation process to optimize the production of CDA and a considerable yield. The *Ri*CDA produced exhibits superior pH and temperature adaptability and stability, has a wide range of substrates, and is easy to purify and prepare. These advantages make it an important candidate for the bioconversion of chitin into high value-added products using a bioenzymatic method. Furthermore, it provides a key element for further research on chitin biorefining.

## Data availability statement

The original contributions presented in the study are included in the article/Supplementary material, further inquiries can be directed to the corresponding authors.

## Author contributions

JX: Writing – review & editing, Writing – original draft, Visualization, Software, Methodology, Investigation, Formal analysis, Data curation, Conceptualization. DoY: Writing – original draft, Visualization, Validation, Methodology, Investigation, Formal analysis, Data curation, Conceptualization. JO: Writing – original draft, Visualization, Validation, Methodology, Data curation, Conceptualization. BL: Writing – review & editing, Methodology, Formal analysis, Data curation, Conceptualization. SL: Writing – review & editing, Validation, Supervision, Project administration, Methodology. DeY: Writing – review & editing, Validation, Supervision, Methodology. HZ: Writing – review & editing, Supervision, Resources, Project administration, Funding acquisition. NS: Writing – review & editing, Validation, Supervision, Resources, Project administration, Methodology, Investigation, Funding acquisition.

## Footnotes

^1^ https://lpsn.dsmz.de/

^2^ http://wh.augct.com/

^3^ https://blast.ncbi.nlm.nih.gov/Blast.cgi

^4^ www.ezbiocloud.net

^5^ https://www.majorbio.com

^6^ http://trna.ucsc.edu/software/

^7^ https://github.com/tseemann/barrnap
