# Supplementary material for: A new strain of Rhodococcus indonesiensis T22.7.1T and its functional potential for deacetylation of chitin and chitooligsaccharides
Source: Front Microbiol. 2024 Jul 24;15:1427143. doi: 10.3389/fmicb.2024.1427143 (PMC11303147; doi:10.3389/fmicb.2024.1427143)
Supplement: Supplementary file 1 [file Table_1.docx]

**Supplemental documents**

**Attached charts**

**
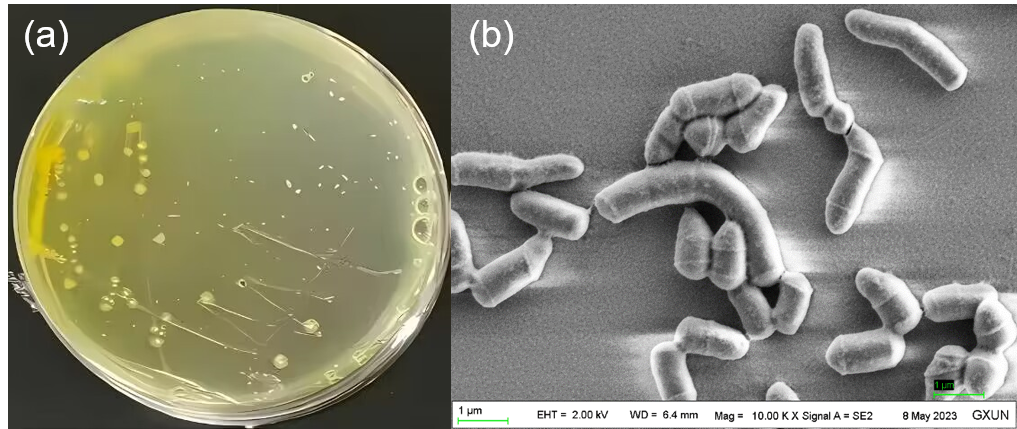
**

Fig S1. Screening of strain T22.7.1 for deacetylation activity and cell morphology. (a) shows strain T22.7.1 cultured on screening medium for 10 days when the medium turned yellow; (b) The scanning electron microscope image of strain T22.7.1^T^ under 100,000-fold magnification.

**
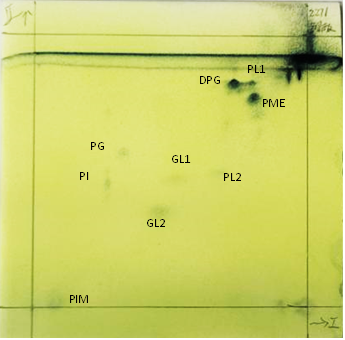

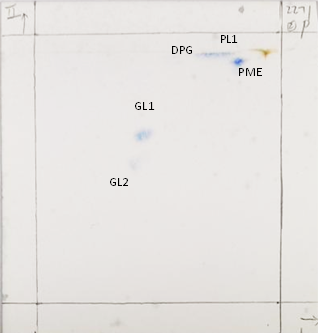
**

**(b)**

**(a)**

**
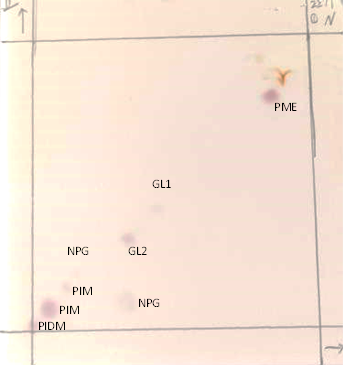

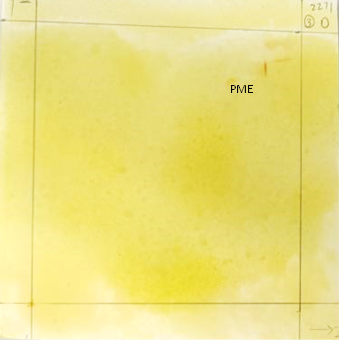
**

**(d)**

**(c)**

**
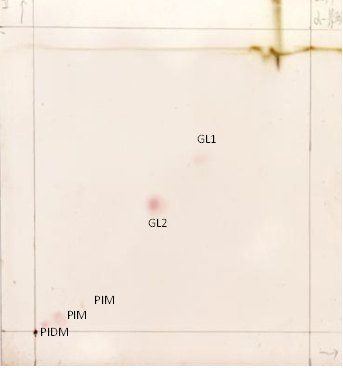
**

**(e)**

**Fig S2.** Analysis of T22.7.1^T^ polar lipid by 2-dimensional TLC. (a) Color result of 10% (w/v) ethanolic molybdophosphoric acid; (b) Color result of molybdenum blue reagent; (c) Color result of ninhydrin reagent; (d) Color result of Dragendorff reagent; (e) Color result of Anisaldehyde reagent.

**Fig S3.** Single factor optimization of medium composition. With the base medium as the control, the order of optimization was carbon source, nitrogen source, inorganic salt and inducer, and the species and then the concentration were optimized first. The error bars are the mean ± SD of three technical replicates for each sample.

**Fig S4.** Single factor optimization of fermentation conditions and fermentation quantity. The error bars are the mean ± SD of three technical replicates for each sample.

**Attached table**

Table S1. Culture characteristics of strain T22.7.1^T^. +, positive; -, negative; w, weakly positive.

| Culture media | Culture characteristics | | |
| --- | --- | --- | --- |
|  | growth | colony color | mobility |
| ISP 1 | + | orange red | - |
| ISP 2 | + | yellowish white/yellow/orange | - |
| ISP 3 | + | orange | - |
| ISP 4 | - | - | - |
| ISP 5 | w | yellowish white/orange | - |
| ISP 6 | + | orange red | - |
| ISP 7 | w | yellowish white/orange | - |
| CA | + | orange | - |
| LB | + | yellowish white/orange/orange red | - |
| SAA | - | - | - |
| GSA | + | orange | - |
| TSA | + | orange red | - |

Table S2. Other physiological and biochemical characteristics of strain T22.7.1^T^

| Property | T22.7.1^T^ | |
| --- | --- | --- |
| M-R test | － |  |
| V-P test | － |  |
| ONPG test | － |  |
| Oxidase | － |  |
| Catalase | ＋ |  |
| H_2_S production | ＋ |  |
| Nitrate reduction | ＋ |  |
| Indole test | － |  |
| Gelatinase | ＋ |  |
| Phenylalanine deaminase | － |  |
| Lysine decarboxylation | ＋ |  |
| Tyrosine hydrolysis test | ＋ |  |
| Growth on sole carbon sources: | |  |
| glucose | ＋ |  |
| lactose | ＋ |  |
| fructose | ＋ |  |
| raffinose | ＋ |  |
| glycerin | ＋ |  |
| Lecithin | － |  |
| Ribose | ＋ |  |
| D-arabinose | ＋ |  |
| trehalose | ＋ |  |
| D-mannose | ＋ |  |

Table S3. Difference of cellular fatty acid content between strain T22.7.1^T^ and its closely related species. 1, T22.7.1^T^; 2, *R. indonesiensis* CSLK01-03^T^; 3, *R. ruber* DSM 43338^T^; 4, *R. electrodiphilus* JC435^T^. -, indicates that the concentration is too low or not detected.

| Type of fatty acids | Content difference of different strains(%) | | | |
| --- | --- | --- | --- | --- |
|  | 1 | 2 | 3 | 4 |
| 10:0 iso | 0.09 | - | - | - |
| 11:0 | 0.23 | - | - | - |
| 10:0 2OH | 0.2 | - | - | - |
| 11:0 iso 3OH | 0.12 | - | - | - |
| 11:0 3OH | ---- | - | - | - |
| 13:0 | 0.11 | - | - | - |
| 14:0 | 3.11 | - | 1.5 | 3.4 |
| 13:0 iso 3OH | 0.29 | - | - | - |
| 15:1 iso F | 0.18 | - | - | - |
| 15:1 iso H/13:0 3OH | 0.09 | - | - | - |
| 15:1 anteiso A | 0.07 | - | - | - |
|  | ---- | - | - | - |
| 15:1 w8c | 0.07 | - | - | - |
| 15:1 w6c | 0.04 | - | - | - |
| 15:1 w5c | 0.22 | - | - | 1.3 |
| 15:0 | ---- | - | 2.9 | - |
| 16:1 w9c | ---- | - | 1.2 | - |
| 16:1 w7c/16:1 w6c | 1.05 | - | - | - |
| 16:1 w6c/16:1 w7c | 10.76 | - | - | 27.9 |
| 16:0 | 26.81 | 37.65 | 27.4 | 25 |
| 16:0 10-methyl | 4.49 | - | 0.6 | 1.3 |
| 17:1 iso I/anteiso B | ---- | - | 15.9 | - |
| 17:1 w9c |  | - | 4.3 | - |
| 17:1 w8c | 1.71 | - | - | 4.8 |
| 17:1 w5c | 0.79 | - | - | - |
| 17:0 | 5.65 | - | 4.3 | 2.9 |
| 17:0 10-methyl | 4.1 | - | 1.8 | 3 |
| 18:1 w9c | 2.65 | 11.88 | 20.7 | 12 |
| 18:1 w7c | 0.49 | - | - | - |
| 18:1 w6c | 0.27 | - | - | - |
| 18:0 | 5.82 | - | 2.4 | 1.7 |
| 18:0 10-methyl, TBSA | 14.97 | 12.05 | 15.6 | 12.2 |
| C18 : 2 ω6/anteiso-C18 : 0 9c | ---- | 10.42 | - | - |
| 19:1 w11c/19:1 w9c | 1.55 | - | 1.3 | - |
| 19:0 cyclo w10c/19w6 | 0.51 | - | - | - |
| 19:0 | 8.87 | - | - | - |
| 20:4 w6,9,12,15c | 3.77 | - | - | - |
| 20:2 w6,9c | 0.33 | - | - | - |
| 20:1 w9c | 0.58 | - | - | - |

Table S4. Genome features of Rhodococcus sp. T22.7.1^T^.

| Items | Description |
| --- | --- |
| Genome size (bp) | 5530537 |
| G+C content (%) | 70.17 |
| GC content in genic regions (%) | 70.42 |
| GC content in intergenic regions (%) | 67.63 |
| Number of protein-coding genes | 5092 |
| Total gene length (bp) | 5042061 |
| Gene/genome (%) | 91.17 |
| Intergenic region length (bp) | 489742 |
| Intergenic length/genome length (%) | 8.85 |
| Number of 5S rRNAs | 1 |
| Number of 16S rRNA | 1 |
| Number of 23S rRNAs | 0 |
| Number of tRNAs | 65 |
| Number of coding genes in the NR | 5063 |
| Number of coding genes in the Swiss-Prot | 3809 |
| Number of coding genes in the Pfam | 4361 |
| Number of coding genes in the COG | 4144 |
| Number of coding genes in the GO | 3854 |
| Number of coding genes in the KEGG | 2471 |

Table S5. Comparison of genomic characterization of strain T22.7.1^T^ with its close relatives.

| characterization | T22.7.1^T^ | *R. indonesiensis* CSLK01-03^T^ | *R. ruber* DSM 43338^T^ | *R. electrodiphilus* JC435^T^ | *R. aetherivorans* 10bc312^T^ |
| --- | --- | --- | --- | --- | --- |
| Genome size (bp) | 5530537 | 5486904 | 5698851 | 5486318 | 6558029 |
| Contig number | 112 | 282 | 3 | 225 | 3 |
| GC content (%) | 70.17 | 70.15 | 70.47 | 70.51 | 70.08 |

Table S6. Comparative analysis of the secondary metabolite synthesis gene cluster of strain T22.7.1^T^ and its close relatives using antiSMASH annotation. 1, T22.7.1^T^; 2, *R. indonesiensis* CSLK01-03^T^; 3, *R. ruber* DSM 43338^T^; 4, *R. electrodiphilus* JC435^T^; 5, *R. aetherivorans* 10bc312^T^. -, indicating that this smBGC was not recognized.

| smBGCs | Gene cluster similarity | | | | |
| --- | --- | --- | --- | --- | --- |
|  | 1 | 2 | 3 | 4 | 5 |
| alkylresorcinol | - | - | - | - | 100% |
| atratumycin | 5% | 10% | 10% | 7% |  |
| azalomycin F3a | - | - | - | - | 8% |
| cinnapeptin | 7% | - | 7% | - | 10% |
| coelichelin | 27% | - | 27% | - | 27% |
| corynecin III/corynecin I/corynecin II | - | 46% | - | - | - |
| ebelactone | - | - | - | - | 8% |
| echoside A/echoside B/echoside C/echoside D/echoside E | 11% | 11% | 11% | 11% | 11% |
| ectoine | 75% | 75% | 75% | 75% | 75% |
| glycopeptidolipid | 5% | 7% | - | - | - |
| heterobactin A/heterobactin S2 | 54% | 36% | 54% | 36% | 54% |
| isorenieratene | 37% | 37% | 37% | 37% | 37% |
| lymphostin/neolymphostinol B/lymphostinol/neolymphostin B | - | 16% | - | - | - |
| madurastatin A2/madurastatin E1/madurastatin F/madurastatin G1/madurastatin A1 | - | 11% | - | - | - |
| methylenomycin A | - | - | - | - | 14% |
| nocardiopsistin A/nocardiopsistin B/nocardiopsistin C | 9% | 9% | 9% | - | - |
| ohmyungsamycin A/ohmyungsamycin B | - | 12% | - | - | - |
| rhizomide A/rhizomide B/rhizomide C | - | 100% | - | - | - |
| rhodochelin | - | - | - | 33% |  |
| SF2575 | 6% | - | 6% | 6% | 6% |
| simocyclinone D8 | - | - | - | 8% |  |
| stenothricin | 18% | - | - | - | - |
| streptozotocin | - | - | - | - | 7% |
| ε-Poly-L-lysine | 100% | 100% | 100% | 100% | 100% |

Table S7, number of protein family members to which strain T22.7.1^T^ and its close relatives belong and the number of genes contained in each protein family predicted by CAZy database (listed in parentheses). 1, T22.7.1^T^; 2, *R. indonesiensis* CSLK01-03^T^; 3, *R. ruber* DSM 43338^T^; 4, *R. electrodiphilus* JC435^T^; 5, *R. aetherivorans* 10bc312^T^. -, indicating that this CAZyme was not recognized.

| CAZyme protein family | Gene Number of CAZyme | | | | |
| --- | --- | --- | --- | --- | --- |
|  | 1 | 2 | 3 | 4 | 5 |
| GHs | 22 (36) | 23 (36) | 24 (37) | 22 (35) | 26 (46) |
| GTs | 16 (49) | 16 (48) | 14 (48) | 15 (47) | 16 (46) |
| PLs | 1 (1) | 0 (0) | 1 (1) | 0 (0) | 0 (0) |
| CEs | 4 (22) | 4 (22) | 3 (21) | 3 (20) | 4 (24) |
| CBMs | 1 (3) | 1 (3) | 1 (3) | 1 (3) | 1 (3) |
| AAs | 3 (10) | 3 (10) | 4 (12) | 3 (11) | 4 (13) |
